# Biomolecular analyses enable new insights into ancient Egyptian embalming

**DOI:** 10.1038/s41586-022-05663-4

**Published:** 2023-02-01

**Authors:** Maxime Rageot, Ramadan B. Hussein, Susanne Beck, Victoria Altmann-Wendling, Mohammed I. M. Ibrahim, Mahmoud M. Bahgat, Ahmed M. Yousef, Katja Mittelstaedt, Jean-Jacques Filippi, Stephen Buckley, Cynthianne Spiteri, Philipp W. Stockhammer

**Affiliations:** 1 Institute for Pre- and Protohistoric Archaeology and Archaeology of the Roman Provinces, Ludwig Maximilian University of Munich, Munich, Germany; 2 Department of Pre- and Protohistory, Eberhard Karls University of Tübingen, Tübingen, Germany; 3 Department of Egyptology, Eberhard Karls University of Tübingen, Tübingen, Germany; 4 Department of Egyptology, Julius-Maximilians University, Würzburg, Würzburg, Germany; 5 The Central Laboratories Network, the National Research Centre, Cairo, Egypt; 6 Packaging Materials Department, the National Research Centre, Cairo, Egypt; 7 Analytical Research Department, Robertet S.A., Grasse, France; 8 BioArCh, University of York, York, UK; 9 Department of Life Sciences, University of Turin, Turin, Italy; 10 Max Planck Institute for Evolutionary Anthropology, Leipzig, Germany

**Keywords:** Science in culture, Lipids, Environmental economics, Bioanalytical chemistry, Interdisciplinary studies

## Abstract

The ability of the ancient Egyptians to preserve the human body through embalming has not only fascinated people since antiquity, but also has always raised the question of how this outstanding chemical and ritual process was practically achieved. Here we integrate archaeological, philological and organic residue analyses, shedding new light on the practice and economy of embalming in ancient Egypt. We analysed the organic contents of 31 ceramic vessels recovered from a 26th Dynasty embalming workshop at Saqqara^1,2^. These vessels were labelled according to their content and/or use, enabling us to correlate organic substances with their Egyptian names and specific embalming practices. We identified specific mixtures of fragrant or antiseptic oils, tars and resins that were used to embalm the head and treat the wrappings using gas chromatography–mass spectrometry analyses. Our study of the Saqqara workshop extends interpretations from a micro-level analysis highlighting the socio-economic status of a tomb owner^3–7^ to macro-level interpretations of the society. The identification of non-local organic substances enables the reconstruction of trade networks that provided ancient Egyptian embalmers with the substances required for mummification. This extensive demand for foreign products promoted trade both within the Mediterranean^8–10^ (for example, *Pistacia* and conifer by-products) and with tropical forest regions (for example, dammar and elemi). Additionally, we show that at Saqqara, *antiu* and *sefet*—well known from ancient texts and usually translated as ‘myrrh’ or ‘incense’^11–13^ and ‘a sacred oil’^13,14^—refer to a coniferous oils-or-tars-based mixture and an unguent with plant additives, respectively.

## Abstract (web summary)

Philological analysis of labels and instructions, together with gas chromatography–mass spectrometry analysis of residues on vessels recovered from a 26th Dynasty embalming workshop at Saqqara, Egypt provide insights into ancient Egyptian embalming practices.

## Main

Ancient Egyptians developed an outstanding ability to protect the human body from decomposition or destruction after death—instigated by the belief that the decomposition of the corpse presented a physical obstacle toward attaining the afterlife^15^. Performed by specialized and learned individuals (ritualist embalmers), embalming was both a chemical and a ritual process^14^. From a chemical perspective, the practice evolved from simple natural preservation (through desiccation), via a proto-embalming treatment during prehistoric times^16^ (around 4,300–3,100 bc), to the sophisticated pharaonic procedures of anthropogenic desiccation (using natron), excerebration, evisceration and the use of antibacterials, antifungals, barrier materials and fixatives^3,15^. This preservation procedure, which could take up to 70 days to complete, ensured the transformation of a vulnerable body into a durable mummy. Embalming also entailed sets of ritualized acts and the recitation of liturgical texts, through which the chemically treated body would be revived and acquired a new identity as a justified or glorified deceased, worthy of living on in the netherworld^17^.

Our present-day knowledge of embalming substances is derived from two main sources: ancient written sources such as embalming papyri^14,18^, and organic residue analyses (ORA) of Egyptian mummies. Substances used in embalming have been named in ancient Egyptian texts and by Greek authors such as Herodotus and Diodorus. However, debates have arisen concerning the specific substances to which these terms correspond^11,15,19,20^. In recent years, ORA has been applied to study residues recovered from mummies and embalming vessels in individual tombs (for example, in ref. ^3^). Although these analyses have successfully identified various substances used in embalming, the roles of these balms in this process as well as the overall procedure have so far remained unclear.

**Fig. 1:**
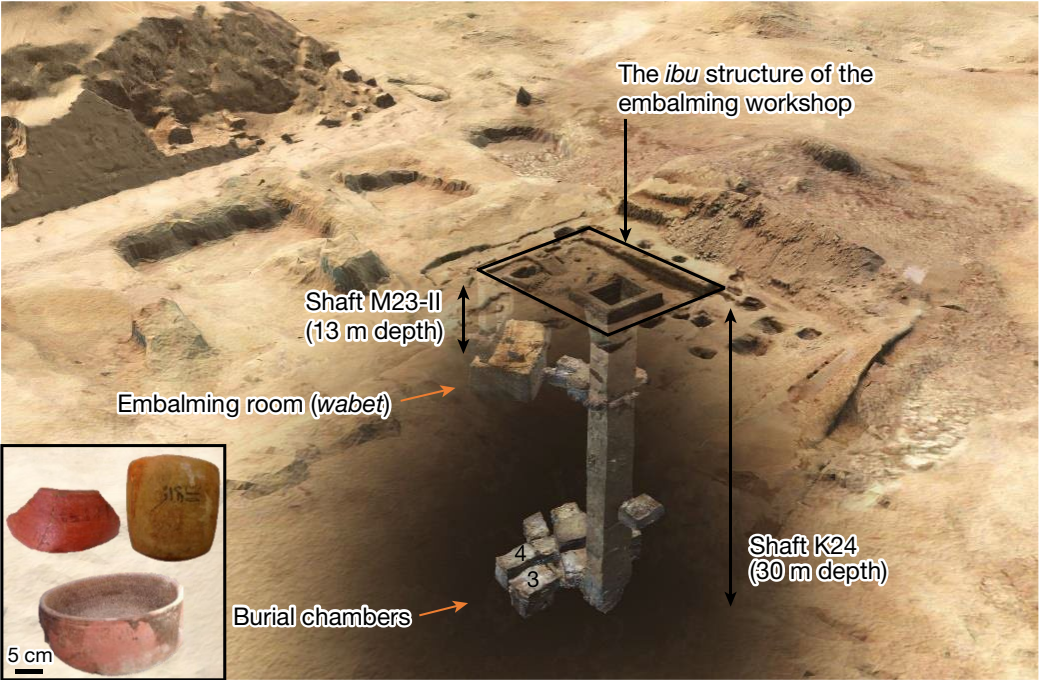
The embalming facilities and burial chambers of the Saqqara complex. Orange arrows show the locations of the investigated vessels. The background image is a digital documentation of the Saqqara complex (copyright M. Lang, Universität Bonn). The two labelled vessels were uncovered in the embalming room and correspond to a ‘red goldfish bowl’ (inscription: ‘*sefet* + dry *antiu*’) and a ‘white clay beaker’ (inscription: ‘to be put on his head’). The unlabelled red bowl with black surface residue was uncovered in the burial chamber loc. 4.

The discovery of embalming facilities at Saqqara presented here reshapes our knowledge and understanding of ancient Egyptian mummification. Dated to around 664–525 bc (26th Dynasty), the embalming workshop is located a few metres to the south of the pyramid of King Unas. It includes a subterranean evisceration facility (the *wabet*), a multifunctional aboveground structure (probably corresponding to the *ibu*) and communal burial spaces^1,2^ (Fig. 1; for a detailed description of the archaeological evidence, see Supplementary Information, section 1). In addition to these structures, a cache of embalming pottery vessels was uncovered in the *wabet* facility. This cache includes a large corpus of potsherds and both broken and complete vessels, with some showing traces of burning as well as drippings of boiled substances on their outer surfaces. Among the finds are 121 beakers and bowls (a total of 59 ‘marl clay beakers’ and 62 ‘red goldfish bowls’; for shapes, refer to Fig. 1) inscribed with Hieratic and Demotic texts providing embalming instructions (for example, ‘to put on his head’ or ‘bandage or embalm with it’) and/or names of embalming substances (for example, ‘*sefet*’ or ‘dry *antiu*’) and sometimes with the title of an administrator of the embalming workshop or the necropolis (Extended Data Table 1). Out of this corpus, we selected 9 beakers and 22 red bowls with the most clearly readable labels for ORA. To establish a possible link with the vessels from the *wabet* facility, we included in our analyses four samples from two burial chambers (locations 3 and 4) at the bottom of the communal burial shaft: two red bowls, one faience cup and one red cylindrical vessel.

## Organic residue analyses

**Extended Data Fig. 1:**
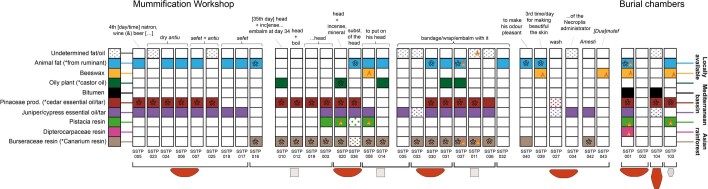
Organic substances identified in the pottery from the Saqqara embalming workshop and communal burial spaces. Flame = molecular markers associated with heat treatment of the substance. Dotted squares = markers present in the substances, but the assemblage is not specific enough.

A wide range of products was identified, including plant oils and tars, resins, and animal fats (details in Extended Data Table 1, Extended Data Fig. 1 and Supplementary Information, section 2).

**Fig. 2:**
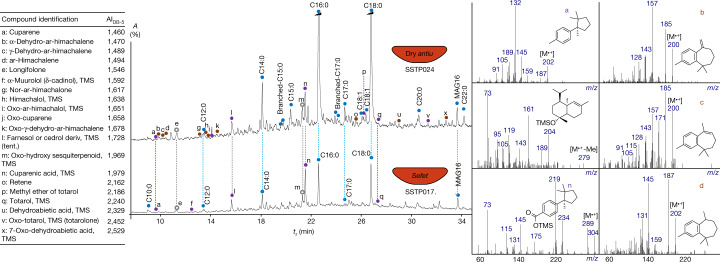
Partial gas chromatograms of organic residue extracts from bowls labelled ‘dry *antiu*’ and ‘*sefet*’. Total ion chromatograms showing the molecular constituents of the essential oil or tar of cedar (brown) and juniper/cypress (purple), and animal fat (blue). Sesquiterpenes and diterpenes are labelled a–z. The prefix SSTP is an identifier for samples from the Saqqara Saite Tombs Project. Right, electron ionization mass spectra (70 eV) of characteristic corresponding sesquiterpenes from coniferous oils or tars. MAG, monoacylglycerol. *A*, abundance; AI_DB-5_, arithmetic retention index; tent., tentative assignment; TMS, trimethylsilyl derivative; *t*_r_, retention time.

Among the group of conifer by-products, juniper or cypress (hereafter juniper/cypress) by-products in the form of essential or fragrant oil or tar were identified in 21 vessels (60%). Their identification is supported by the association of totarol derivatives and cuparene-related sesquiterpenes^21,22^ (Fig. 2). The cedar oil or tar is the second most commonly detected product in the Saqqara vessels (19 vessels (54%)). Its presence is indicated by the predominance or the equivalence of low molecular weight sesquiterpenoids of the himachalene series over the characteristic diterpenes of the abietane family^22–24^ (Fig. 2).

**Fig. 3:**
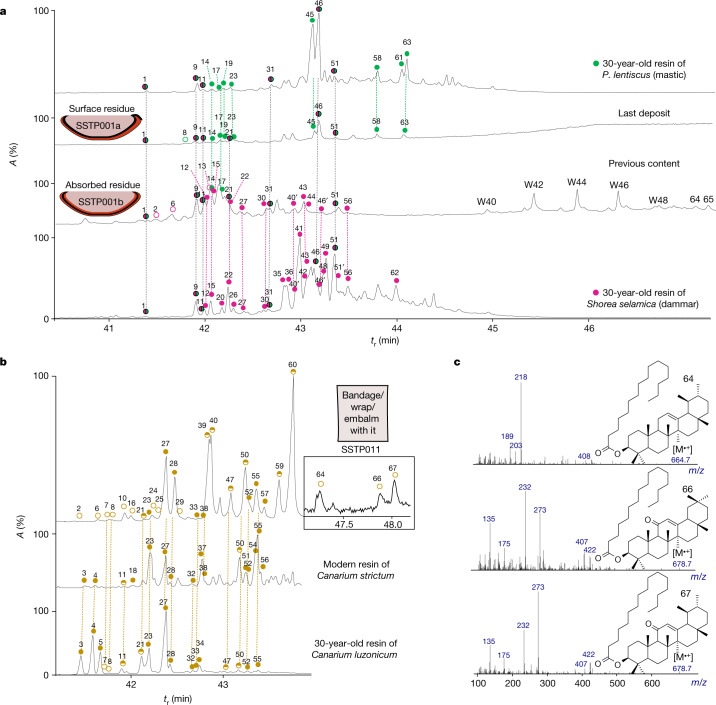
Partial gas chromatograms showing the molecular constituents of *Pistacia* resin, dammar and beeswax. **a**,**b**, Total ion chromatograms showing the molecular constituents of *Pistacia* resin (visible surface residue), dammar and beeswax (absorbed residue) (**a**) and elemi (**b**). **c**, Electron ionization mass spectra (70 eV) of triterpenic palmitates. Green circles indicate markers of *Pistacia* resin, pink circles indicate markers of dammar resin and yellow circles indicate markers of elemi. Filled circles are biomarkers present in fresh resin, empty circles are degradation markers linked to natural oxidation and/or heat treatment and half-filled circles are biomarkers and/or degradation markers. Numbers prefixed with W are the number of carbon atoms in the long-chain esters associated with the corresponding peak. Triterpenes are labelled numerically as follows: 1, 28-norolean-12, 17-dien-3-one; 2, olean-9(11),12-dien-3-one; 3, 3-epi-β-amyrin; 4, 3-epi-α-amyrin; 5, 3-epi-lupeol; 6, olean-9(11)-en-3-one; 7, urs-9(11),12-dien-3-one; 8, olean-9(11),12-dien-3-ol; 9, nor-β-amyrone (28-norolean-12-en-3-one); 10, α-amyrenone isomer (urs-9(11)-en-3-one); 11, β-amyrenone; 12, dammaradien-3-one; 13, olean-18-en-3-one; 14, 28-noroleandien-3-one or 28-norursdien-3-one (tent.); 15, nor-α-amyrenone (28-norurs-12-en-3-one); 16, urs-9(11),12-dien-3-ol; 17, 28-norolean-17-en-3-one; 18, olean-3,12-dien-16-ol (dehydroxymaniladiol); 19, oleandienol; 20, nor-β-amyrin (28-norolean-12-ene-3-ol); 21, α-amyrenone; 22, dammaradien-3-ol (3β-hydroxy-20,24-dammarediene); 23, β-amyrin; 24, lupenone; 25, olean-9(11),12-dien-3-yl acetate; 26, nor-α-amyrin (28-norurs-12-ene-3-ol); 27, α-amyrin; 28, lupeol; 29, ursa-9(11),12-dien-3-yl acetate; 30, δ-amyrenone (olean-13(18)-en-3-one); 31, noroleanenol or norursenol (tent.); 32, maniladiol (olean-12-ene-3,16-diol); 33, 11-oxo-β-amyrin epi-isomer (tent.); 34, 11-oxo-α-amyrin epi-isomer (tent.); 35, dammarenolic acid; 36, shoreic acid; 37, lupeol isomer; 38, brein (urs-12-ene-3,16-diol); 39, β-amyrin acetate; 40, α-amyrin acetate; 40′, 20,24-epoxy-25-hydroxydammaren-3-one; 41, hydroxydammaradienone (tent.); 42, oleandien-28-ol (tent. erythro-3-en-28-ol); 43, hydroxydammarenone; 44, oleanonic aldehyde; 45, moronic acid; 46, oleanonic acid; 46′, 20,24-epoxy-25-hydroxydammaren-3-ol; 47, 11-oxo-β-amyrenone; 48, hydroxydammarenol; 49, oleanol derivative or ursol derivative; 50, 11-oxo-α-amyrenone; 51, oleanolic acid; 51′, ursonic acid (3-oxours-12-en-28-oic acid); 52, 11-oxo-β-amyrin; 53, ursolic aldehyde; 54, oleanolic aldehyde; 55, 11-oxo-α-amyrin; 56, ursolic acid; 57, lupane derivative (tent. canaric acid); 58, isomasticadienonic acid; 59, 11-oxo-β-amyrin acetate; 60, 11-oxo-α-amyrin acetate; 61, 11-oxo-oleanonic acid; 62, hydroxy oleanolic acid; 63, masticadienonic acid; 64, α-amyrin palmitate (urs-12-en-3-yl palmitate); 65, oxo-oleanene palmitate; 66, 11-oxo-β-amyrin palmitate; 67, 11-oxo-α-amyrin palmitate.

With regard to angiosperm resins, we identified elemi in at least 15 vessels (43%) (Extended Data Fig. 1) on the basis of the combination of lupeol and α- and β-amyrin derivatives (Fig. 3). This assemblage is commonly associated with resin of Burseraceae, particularly that of *Canarium*^25–29^ (also known as elemi) (Extended Data Table 2). *Bursera* and *Protium* resins could be excluded as they occur mainly in Central and South America^21,30^. α- and β-11-keto amyrins were identified in the 15 vessels (sometimes together with their acetate derivatives), in two cases together with traces of brein (urs-12-ene-3,16-diol). These biomarkers are documented in elemis from the Asian rainforest^25,26,29^ but elemis from the African rainforest should not be excluded (Extended Data Table 2). Finally, the compounds, olean-9(11),12-dien-3-ol and urs-9(11),12-dien-3-ol were detected in 14 samples (Fig. 3 and Extended Data Table 2). These were previously identified in artificially aged elemis from Manila and Mexican copal^28^. In addition, *Pistacia* resin was detected in five vessels (14%). The identification was on the basis of the presence of characteristic biomarkers^5,8,31^ (for example, moronic, oleanonic, isomasticadienonic and masticadienonic acids) (Fig. 3). Triterpenic markers of heat treatment^8^ were also identified in four vessels (Extended Data Fig. 1). A Dipterocarpaceae resin, commonly known as dammar, was detected in one red bowl from burial chamber, location 4. This resin is characterized by a broad assemblage of triterpenic markers from dammarane, nor-ursane and oleanane families. Although some of these biomarkers are ubiquitous, the co-occurrence of dammaradien-3-ol, nor-α-amyrone, δ-amyrone and oxidation products such as 20,24-epoxy-25-hydroxydammaren-3-ol is a convincing argument for the identification of dammar. To our knowledge, these compounds have not been found together in any other resin^31–34^ (Fig. 3).

**Extended Data Fig. 2:**
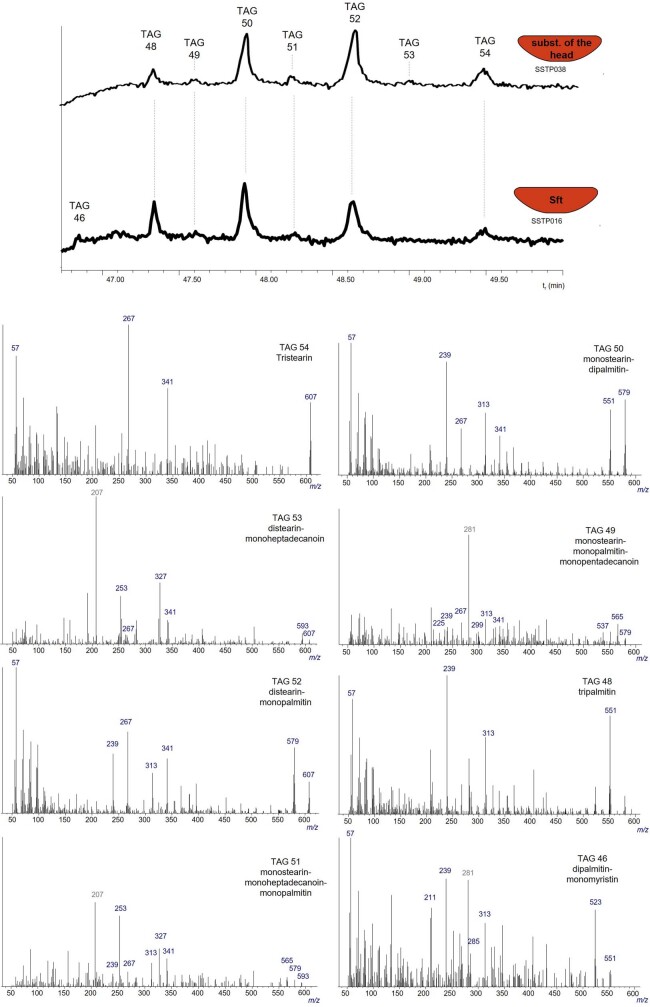
Characteristic biomarkers of ruminant fat. Partial Total Ion Chromatograms and mass spectra (EI, 70 eV) showing even and odd saturated triacylglycerols (TAGs).

**Extended Data Fig. 3:**
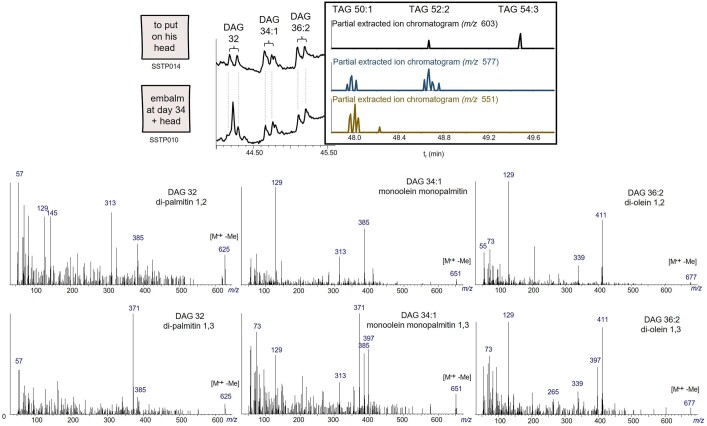
Characteristic biomarkers of plant oil. Partial Total Ion Chromatograms and mass spectra (EI, 70 eV) showing unsaturated di- and triacylglycerols (DAGs and TAGs).

**Extended Data Fig. 4:**
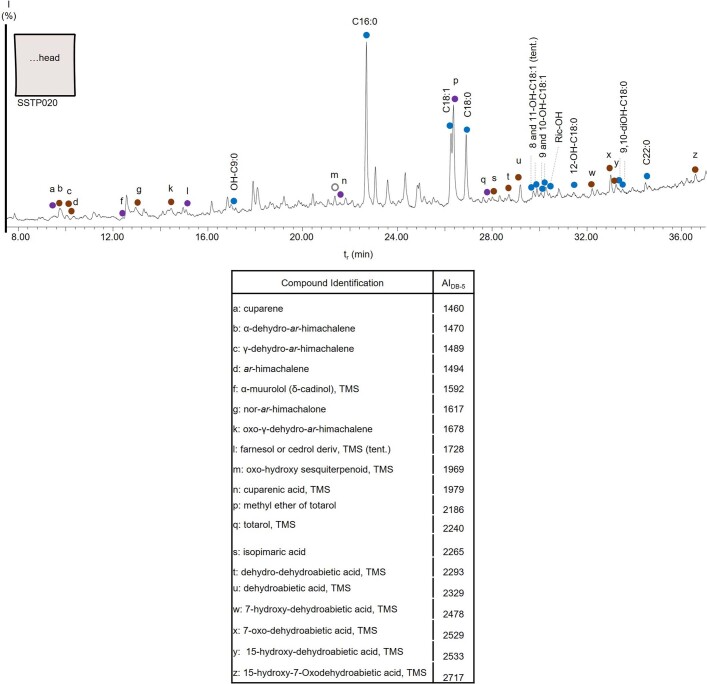
TIC showing the molecular constituents of castor oil and possibly other plant oils (blue), oil/tar of cedar oil/tar (brown) and juniper/cypress (purple) in a white beaker labelled “head”. Cx:y = fatty acid with x carbon atoms and y representing the number of unsaturation; OH-C = hydroxy fatty acid; diOH-C = dihydroxy fatty acid; Ric-OH = ricinoleic acid. AI_DB-5_ = arithmetic retention index.

Animal fat was detected in 18 vessels (51% of vessels). Its presence was indicated by a narrow distribution of saturated triacylglycerols (TAGs) (46:0 to 54:0 (carbon atoms:unsaturated carbon–carbon bonds)) and diacylglycerols^35^ (32:0 to 36:0). Furthermore, traces of saturated TAGs with an odd number of carbon atoms (53:0, 51:0 and 49:0), which are characteristic of ruminant animal fats^36^, were identified in 7 vessels (Extended Data Figs. 1 and 2). Plant oils were detected in 5 vessels (14%). In 4 of them, a plant oil, type olive (although degraded argan or hazelnut oils cannot be excluded) was indicated by the specific distribution of unsaturated TAGs (54:3, 52:2, 50:1) and diacylglycerols^37^ (34:1, 36:2 and 32:0) (Extended Data Fig. 3). The detection of ricinoleic acid together with a substantial amount of oleic acid and its mono- and dihydroxylated degraded markers in one beaker suggests that it may have contained castor oil, possibly mixed with other oils^38–40^ (Extended Data Fig. 4). Although ricinoleic acid has also been associated with the activity of ergot fungi on Gramineae^41^, the castor oil hypothesis remains the most plausible in the Saqqara context, where embalming vessels were dedicated to the preparation of antiseptic and antifungal substances for mummification. Beeswax was identified in 5 vessels (14%) by the presence of its characteristic even-numbered fatty acids (22:0 to 28:0, with 24:0 being the most important) and long-chain (C_40_ to C_48_) palmitic esters^5,42^ (Fig. 3).

**Extended Data Fig. 5:**
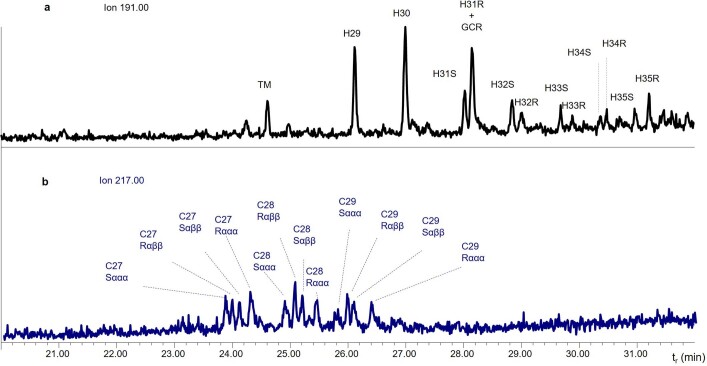
Bitumen biomarkers. Ion extract chromatogram (m/z 191 and 217) showing **a** the hopanes and **b** the steranes. Hopanes = TM: 17α(H), 22,29,30-trisnorhopane; H29: 17α(H),21β(H)-norhopane (C29); H30: 17 α (H),21β(H)-hopane (C30); H31S: 22S-30-homohopane (C31); H31R: 22R-30-homohopane (C31); GCR: gammacerane; H32S: 22S-30,31-bishomohopane (C32); H32R: 22R-30,31-bishomohopane (C32); H33S: 22S-30,31,32-trishomohopane (C33); H33R: 22R-30,31,32-trishomohopane (C33); H34S: 22S-30,31,32,33-tetrakishomohopane (C34); H34R: 22R-30,31,32,33-tetrakishomohopane (C34); H35S: 22S-30,31,32,33,34-pentakishomohopane (C35); H35R: 22R-30,31,32,33,34-pentakishomohopane (C35). Steranes = C27Sααα: 20S-5α(H),14α(H),17α(H)-cholestane (C27); C27Rαββ: 20R-5α(H),14β(H),17β(H)-cholestane (C27); C27Sαββ: 20S-5α(H),14β(H),17β(H)-cholestane (C27); C27Rααα: 20R-5α(H),14α(H),17α(H)-cholestane (C27); C28Sααα: 20S-5α(H),14α(H),17α(H)-ergostane (C28); C28Rαββ: 20R-5α(H),14β(H),17β(H)-ergostane (C28); C28Sαββ: 20S-5α(H),14β(H),17β(H)-ergostane (C28); C28Rααα: 20R-5α(H),14α(H),17α(H)-ergostane (C28); C29Sααα: 20S-5α(H),14α(H),17α(H)-stigmastane (C29); C29Rαββ: 20R-5α(H),14β(H),17β(H)-stigmastane (C29); C29Sαββ: 20S-5α(H),14β(H),17β(H)-stigmastane (C29); C29Rααα: 20R-5α(H),14α(H),17α(H)-stigmastane (C29).

Bitumen was found in two vessels recovered from the burial chambers at locations 3 and 4, based on the characteristic hopanes and steranes^3,23,43,44^ (Extended Data Fig. 5). Its chemical composition suggests that it originated from the Dead Sea^23^ (Supplementary Information, section 2).

Finally, we identified molecular markers of recipes that involve the mixing and heating of resinous substances with fat or oil in three vessels (triterpenic palmitates; Supplementary information 2 and ref. ^45^). Elemi was prepared together with animal fat and/or plant oil in two beakers and dammar was prepared with beeswax and/or animal fat in a bowl (Fig. 3 and Extended Data Fig. 1).

## Treatment of the body in the workshop

The inscriptions on the vessels of the Saqqara workshop contain instructions for the treatment of specific body parts, especially the head, and for the preparation of linen bandages. Some of these treatments involved the preparation and application of several mixtures.

**Fig. 4:**
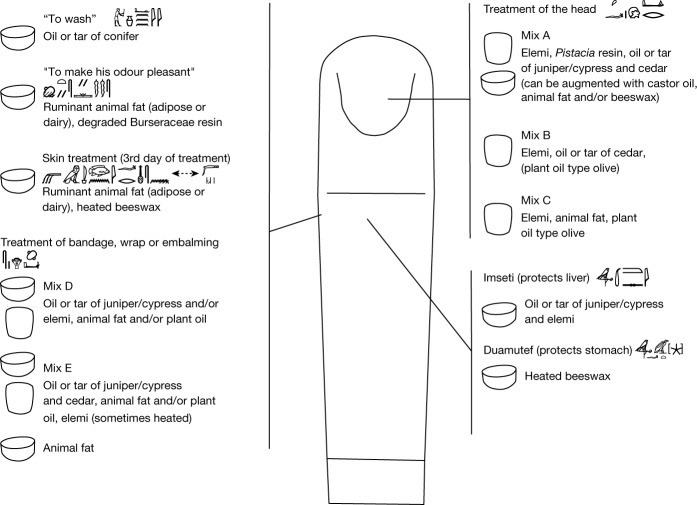
Organic contents of vessels providing embalming instructions. Organic substances and/or mixtures identified in the pottery and the inscriptions associated with these vessels. Mummy drawing copyright S. Lucas.

Eight vessels are inscribed with instructions for the treatment of the head. Our samples show that the embalmers used three different mixtures (mixtures A, B and C in Fig. 4 and Extended Data Fig. 1), which can include elemi, *Pistacia* resin an oil or tar of juniper/cypress and cedar, animal fat, beeswax, probably castor oil, and a plant oil (type olive). To our knowledge, the use of elemi and oil or tar of juniper/cypress for embalming the head has not previously been reported. However, previous ORA studies of early mummies from the first millennium bc suggest, in accordance with our results, that castor oil and *Pistacia* resin were used specifically for the treatment of the head^6,40,46^. Beeswax, Pinaceae by-product, and fat or oil were additionally used for different parts of the body^3–6^.

We extracted samples from eight vessels (four beakers and four red bowls) with labels indicating for ‘wrapping or embalming with it’, which were probably used for preparing mummy linen bandages. The organic contents of seven vessels were mixtures (mixtures D and E; Fig. 4 and Extended Data Fig. 1), and one bowl contained only animal fat. Mixture E was the most frequently detected (five vessels) and consisted of oil or tar of juniper/cypress and cedar, animal fat and/or plant oil and elemi. In two of these vessels, we additionally identified heating markers of elemi resin together with fat or oil. Previous studies of mummy bandages from the 4th millennium bc and later provide evidence for the use of fat or oil and conifer by-product in most of the balms, but none for the use of elemi^3–6,16^. However, one sample from the 1st millennium bc was treated with a mixture including fat or oil, a conifer by-product and a triterpenic resin resembling mixture E^5^. Previous studies have provided evidence that bitumen and beeswax were regularly incorporated into balms for bandages during this period^3–5,7^. However, neither of these substances were detected in vessels used for the mummy bandages at Saqqara (although the limited amount of residue absorbed prevented the application of targeted methods^6,47^). Instead, we found two new substances—elemi and juniper/cypress.

Six other sherds provided information on substances used for washing the body, reducing bodily odour and softening the skin, as well as a recipe for the treatment of the liver and another for the stomach. The bowl labelled with ‘to wash’, contained markers of oil or tar of conifer, and the bowl inscribed with ‘to make his odour pleasant’ showed evidence of ruminant animal fat (adipose or dairy) and degraded Burseraceae resin (Extended Data Table 2). In the vessel with inscriptions related to the treatment of the skin, which may have occurred on the third day of embalming (Extended Data Table 1), we identified a mixture of animal ruminant fat (adipose or dairy) combined with heated beeswax.

Two of the sampled vessels were inscribed: one with the name of the god Imseti, who protects the liver, and the other with the god Duamutef, who protects the stomach. One of these vessels (Imseti/liver) contained a mixture of oil or tar of juniper/cypress and elemi, whereas the other (Duamutef/stomach) contained only heated beeswax (potentially similar content of two 26th Dynasty canopic jars is described in ref. ^7^).

Another bowl was inscribed with the title of an administrator of the embalming workshop and the necropolis—the seal bearer—who carried out specific embalming procedures, related mainly to the treatment of the head^14^. This vessel yielded fat or oil and oil or tar of juniper/cypress, which is identical to mixture D, for treating linen bandages and which could have been used to wrap the head.

## Embalming vessels in the burial chambers

The embalmers of the workshop also provided additional services, including the burial of the deceased in communal burial spaces^1^. We analysed four vessels from two communal burial chambers (locations 3 and 4) to evaluate similarities and differences among the substances used during burial.

One bowl from location 4 was used multiple times and for different substances. A visible black residue lining its surface was identified as a pure heated *Pistacia* resin. However, the ceramic sample taken from its inner wall showed markers of oils or tars of cedar and juniper/cypress, bitumen and dammar mixed with beeswax and/or animal fat. This points to the complex and extended usage of the vessel, used first to prepare the different substances (ceramic impregnation) and subsequently to contain a heated *Pistacia* resin (last deposit).

From burial chamber location 3, we analysed a small faience cup and a red cylindrical pottery vessel. The cup still contained a cake-like substance, consisting of oil or tar of cedar, animal fat, heated *Pistacia* resin and heated beeswax. The cylindrical vessel contained oil or tar of cedar and possibly of juniper/cypress as well as bitumen and a fat or oil.

With the exception of the dammar and bitumen, all the substances detected in the vessels recovered from the burial chambers matched those identified in the embalming workshop.

## Properties and management of substances

These results suggest that the embalmers used the substances for their specific biochemical properties, as *Pistacia* resin, elemi, dammar, oils, bitumen and beeswax have antibacterial or antifungal and odoriferous properties, and thus help to preserve human tissue and reduce unpleasant smells^4,33,42,44^. Animal fat, plant oil and beeswax were also essential ingredients in recipes for the treatment of different body parts, as well as in ointments used to moisturize the skin^48^. Finally, the hydrophobic and adhesive properties of tars, resins, bitumen and beeswax were useful to seal skin pores, exclude moisture and to treat linen wrappings. The colour or appearance of these products may also have been desirable^4^.

The embalming substances identified point to the existence of a management system of bio-products, from harvest, transportation, transformation and application. For example, obtaining plant oil and animal fat necessitate an extraction system, and the production of wood tar (pyrolysis) or oil (steam distillation) involves thermal processing and the specific controlled management of the raw material^49^. In addition, the thermal treatment of substances (such as *Pistacia* resin and beeswax) and the subsequent production of recipes (for example, those based on elemi and dammar resins) required specialized knowledge, technical skills and tools to obtain balms with the desired properties. Our results demonstrate that the embalmers indeed carried out activities that require specific know-how and benefited from institutional organization.

## *Antiu* and *sefet*

An important challenge for understanding Egyptian embalming practices on the basis of textual sources has always been the translation of substance-related terms^20^. Lexicographically, *antiu* has tentatively been associated with myrrh on the basis of philological conjectures^11–13^. However, five vessels from the embalmers’ workshop that carry the label *antiu* yield a mixture of oil or tar of cedar and juniper/cypress together with animal fat (Extended Data Fig. 1; the use of cedar and/or juniper/cypress oil in ancient Egypt is described in refs. ^22,23,46,50,51^). The labels indicate that *antiu* could have been used alone in dry form or mixed with *sefet*. However, in all cases we find markers of a mixture of coniferous volatile products with animal fat. This strongly suggests that *antiu* is a product that was purposefully manufactured by the embalmers and whose preparation entails the transformation of at least two different coniferous oils or tars and then mixing them with animal fat. In the Saqqara context, translations of *antiu* as a raw material such as myrrh can be excluded.

In Egyptology, *sefet* is usually described as an unidentified oil^12,13,48^. It was one of the ‘7 sacred oils’ that were used in embalming and the ‘opening of the mouth’ ritual^13,14^. In three vessels from the embalmers’ workshop with the label ‘*sefet*’, we identified markers of animal fats, which were mixed in two of these vessels with oil or tar of juniper/cypress. The third vessel contained the markers of ruminant fat (adipose or dairy) with elemi. This indicates that, at least at Saqqara, *sefet* was a scented unguent (fat-based formula) with plant additives, particularly Cupressaceae or Burseraceae by-products. It is possible that the scented *sefet* unguent was also prepared with other plant oils. Moreover, its composition may have evolved over time^14,46,51^.

## Egyptian mummification and the world economy

**Fig. 5:**
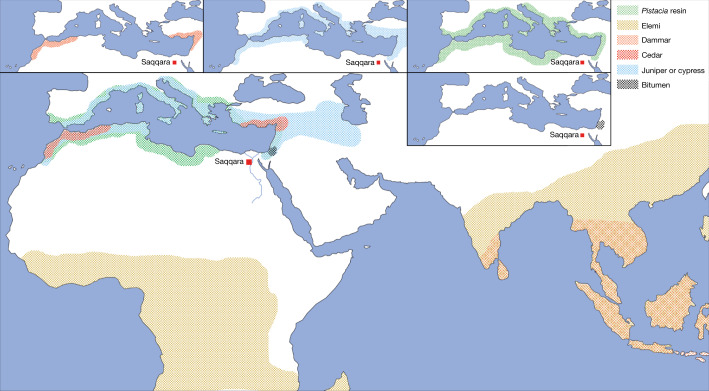
Potential origins of imported bioresources at Saqqara complex. Coloured areas indicates the potential origins of the raw materials that were used for the preparation of balms and the mummification processes at Saqqara. Map copyright S. Lucas.

**Extended Data Fig. 6:**
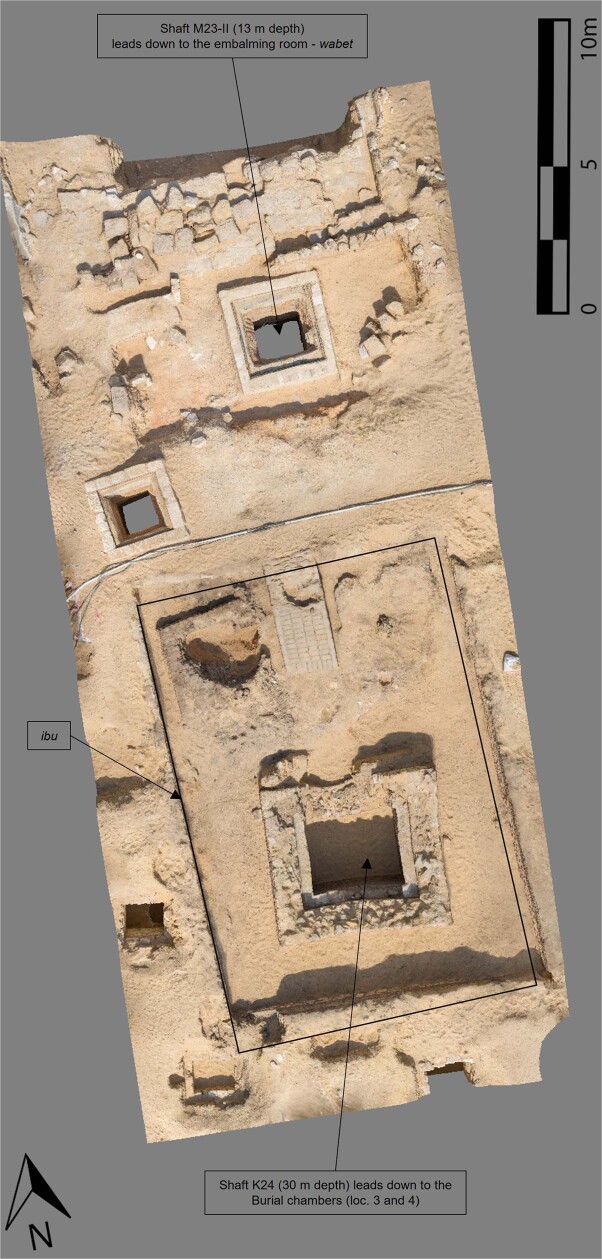
Saqqara complex and location of the investigated vessels in the embalming facilities and burial chambers. Background = digital documentation of Saqqara complex. Copyright M. Lang, Universität Bonn.

**Extended Data Fig. 7:**
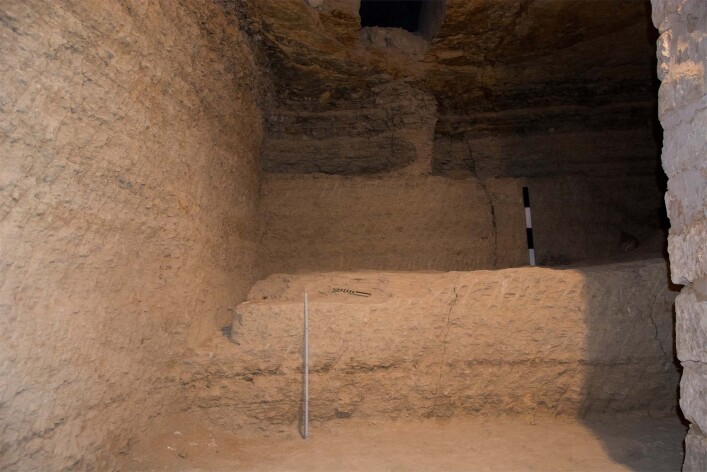
Photograph of Shaft M23-II (left side). Embalming workshop/cachette room with ledge-like bed and drainage channel, looking east. Copyright SSTP.

**Extended Data Fig. 8:**
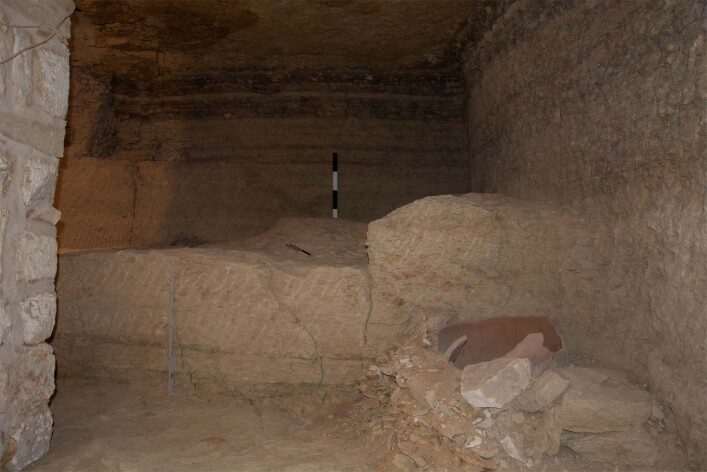
Photograph of Shaft M23-II (right side). Embalming workshop/cachette room with the ledge-line bed and the large fumigation vessel on the right side, looking east. Copyright SSTP.

The majority of the substances used at the Saqqara workshop were imported—many of them from a considerable distance. The Saqqara context (Extended Data Figs. 6, 7 and 8) provides only a glimpse into the trade and exchange systems required to run a comprehensive embalming industry^3,15,52^. These findings confirm the known pattern of the diversification and complexification of embalming practices after around 1000 bc^3,5^. The origin of the different substances provides evidence for an almost global network (Fig. 5). The bitumen identified in Saqqara most probably originated from the Dead Sea, confirming previous findings that the asphalt from this region was exported to Egypt in the first millennium bc specifically for mummification^4,53^. *Pistacia* trees producing high yields of resin (*Pistacia lentiscus* or *Pistacia terebinthus*), olive trees, cedar, juniper and cypress are absent in Egypt^8,11,21,30^, but grow in different locations in the Mediterranean basin (Fig. 5). The related by-products were also imported, most probably from the Levant (for example, *Cedrus libani*), which had important trade networks with Egypt^8–10^.

Although intensified trade networks and cross-cultural exchanges are well-documented for the regions of the Mediterranean basin, the Saqqara workshop provides additional evidence for long-distance trade networks via the vivid Indo-Mediterranean trade routes, which seem to have existed since the 2nd millennium bc^54^. This is particularly true for resins, which are endemic to rainforests. *Canarium* species, which produce elemi, are distributed in both Asian and African rainforests^21,30^, whereas dammars are harvested from Dipterocarpaceae trees that grow exclusively in Asian tropical forests^21,30^. Thus, it is possible that elemi reached Egypt by the same route as dammar^55^. Consequently, the embalming and funerary services of the 7th century bc Saqqara workshop kept the demand for such biomaterials from distant lands active and supported the flourishing of international trade networks connecting Egypt with the eastern Mediterranean in addition to Asian and possibly African rainforests.

## Conclusion

We have identified several specific mixtures used for embalming the head or wrapping the body. The mummification specialists seem to have been aware of both the chemical properties and the bioactivity of the substances used and to have obtained complex knowledge about the preparation of different balms of particular ingredients. We identified *antiu* and *sefet* as mixtures of different fragrant oils or tars and fats. *Antiu* should be less restrictively designated—that is, not exclusively as myrrh or incense. Egyptian mummification was built upon and fostered long-distance exchange and routes, including imports from the Mediterranean basin as well as Asian and possibly African rainforest regions.

## Methods

Upon the discovery of the embalming vessels of the Saqqara workshop, a multinational team of researchers from the Universities of Tübingen and the Ludwig Maximilian University of Munich (Germany), and the National Research Centre (NRC) of Cairo (Egypt) was formed. Vessels were sampled on site at Saqqara and samples were delivered to the NRC laboratories for extraction and analyses.

### Sample treatment before GC–MS analyses

ORA was carried out at the NRC, Chromatographic Laboratories Network, Giza, Egypt. One gram of pottery powder was drilled out from the inner walls of the vessel (layer 2), following cleaning of its surfaces in order to remove any exogenous lipids. The characterization of the lipid constituents present was based on the analytical results obtained from layer 2. The ceramic powder collected during surface cleaning (layer 1) was retained for potential additional analysis. Powdered sherds were solvent-extracted (dichloromethane:methanol, 2:1 by volume) by ultrasonication to target lipid and resin compounds following established protocols^56^. 50% of the total lipid extract were trimethylsilylated (40 °C for 20 min) using *N,O*-bis(trimethylsilyl)trifluoroacetamide (BSTFA) (50 μl) and a catalytic reagent (pyridine) (4 μl) before analysis by gas chromatography–mass spectrometry (GC–MS).

Modern and aged (30-year-old) angiosperm resins, which included *Pistacia*, dammars, frankincense, elemi and myrrh (Extended Data Table 3) were ground, then extracted by ultrasonication in dichloromethane (1 mg ml^−1^) and trimethylsilylated following established protocols^49^.

### Gas chromatography and GC–MS analyses

The analysis of trimethylsilylated samples was performed by GC–MS using an Agilent 7890B GC system and Agilent 5977 MSD.

The analyses were carried out using helium as a carrier gas, with a split/splitless injection system (SSL), operating in the splitless mode with a flow rate of 3.0 ml min^–1^ of helium and a constant pressure at the head of the column of 8.6667 psi. Samples were analysed using an Agilent J&W DB-5HT-column (15 m × 0.32 mm internal diameter; 0.1 μm film thickness). The temperature of the oven was set at 50 °C for 1 min then ramped to 100 °C at 15 °C min^–1^, then to 240 °C at 4 °C min^–1^ and to 380 °C at 20 °C min^–1^ (held isothermally for 7 min). The inlet temperature was set at 300 °C. Mass spectra were acquired using electron ionization at 70 eV and obtained by scanning between *m*/*z* values 50 and 950. The interface and the ion source temperatures were 300 °C and 230 °C, respectively.

Some samples composed of triterpenoid markers and determined to be free of high molecular weight components (absence of wax esters, TAGs, triterpene palmitate) by the conditions described above, were analysed using an Agilent J&W DB-5MS column (30 m × 0.25 mm internal diameter; 0.25 μm film thickness). The inlet temperature was fixed at 300 °C. The oven temperature was ramped from 50 °C (held isothermally for 1 min) to 150 °C at 10 °C min^–1^, and then increased to 320 °C at 4 °C min^–1^ (held isothermally for 15 min). The analyses were carried out using helium as a carrier gas, with a flow rate at 2.0 ml min^–1^ and the operating in the splitless mode with a purge flow of 3.0 ml min^–1^ and a split ratio of 3:1. Mass spectra were acquired using electron ionization at 70 eV. The mass range was scanned for *m*/*z* 50–950. The ion source temperature was set at 230 °C and the transfer line at 250 °C.

Chromatograms and mass spectra were matched against authentic standards (lupeol, lupenone, α- and β-amyrin, saturated and unsaturated triglycerides, fatty acids, *n*-alkanes)^8,22,28,31,57–59^ and the National Institute of Standards and Technology (NIST) library^60^.

Retention indices were calculated based on a series of straight chain hydrocarbons from 7 to 40 carbons and were also used to confirm the identification of sesquiterpenes and diterpenes. The arithmetic retention indexes (AI) used in ref. ^59^ were computed as: AI(*x*) = 100z  + 100[(RT(*x*) − RT(*P*_z_))/(RT(*P*_z_ + 1) − RT(*P*_z_))], according to Van den Dool and Kratz^61^; *x*, analyte; RT, retention time; *P*_*z*_ are paraffins (*n*-alkanes) with *z* carbon atoms.

### Reporting summary

Further information on research design is available in the Nature Portfolio Reporting Summary linked to this article.

## Online content

Any methods, additional references, Nature Portfolio reporting summaries, source data, extended data, supplementary information, acknowledgements, peer review information; details of author contributions and competing interests; and statements of data and code availability are available at 10.1038/s41586-022-05663-4.

### Supplementary information

Supplementary Information The Supplementary Information contains Supplementary Sections 1 and 2 which present a detailed description of the archaeological context of the Saqqara complex (embalming workshop and ancient Egyptian embalming facilities) and of the results of the organic residue analyses (molecular assemblages associated with the identified substances).

Reporting Summary

## Figures and Tables

**Extended Data Table 1:**
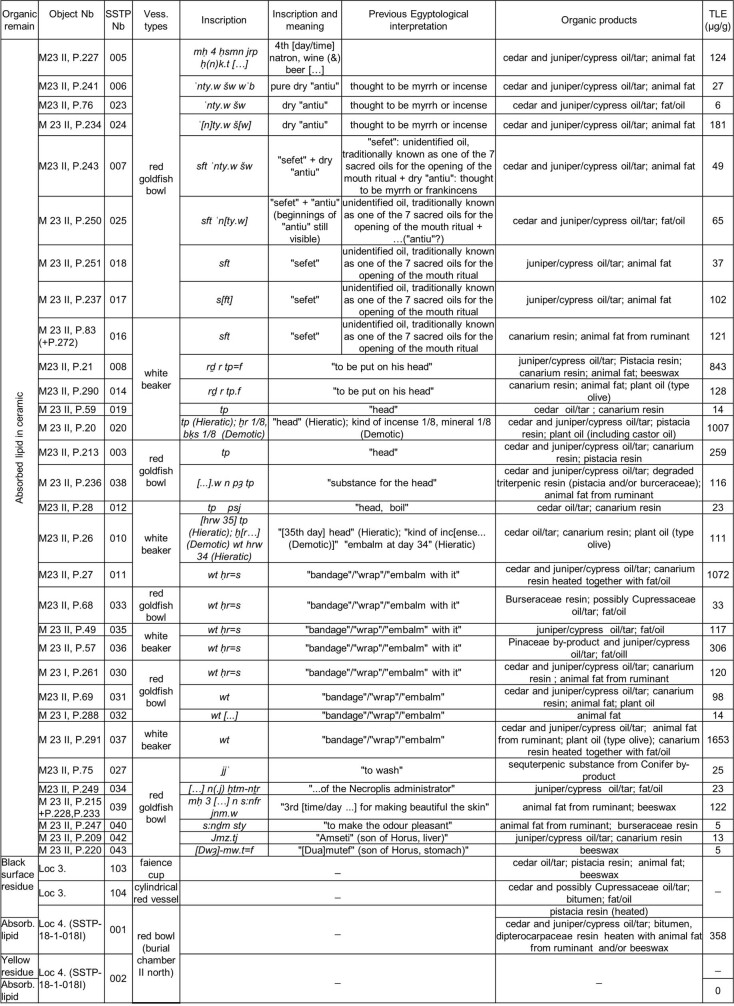
List of samples including the vessels’ inscription, ORA results and quantification

Vessels are currently stored in the Deposit room-magazine at the site of Saqqara, Egypt. TLE = Total lipid extract/g of ceramic.

**Extended Data Table 2:**
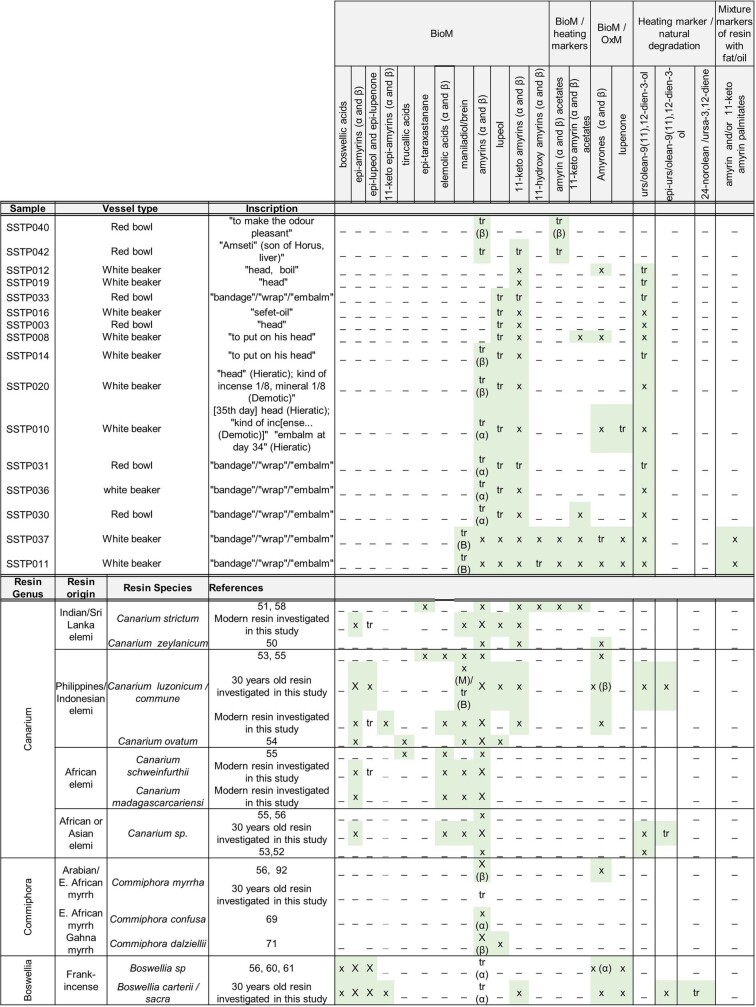
Synthesis of the triterpenoid components of Burseraceae resins and comparison with the products identified in Saqqara

Numbers correspond to references listed in the Supplementary information. BioM = biomarkers and OxM = oxidation markers.

**Extended Data Table 3:**
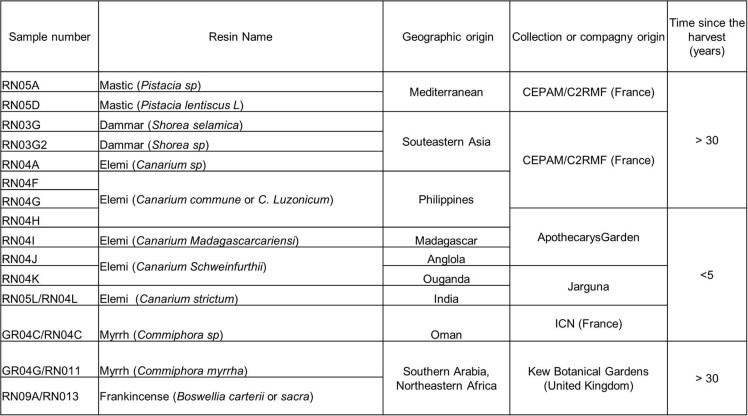
Modern and aged angiosperm resins used as references

List of modern and aged (30 years old) angiosperm resins which have been analysed under similar conditions for comparison with the archaeological samples.

## Data Availability

All information on the samples and the data generated and analysed in this study is included in the manuscript, supplementary information files and Extended Data files.
