# Supplementary material for: Biomolecular analyses enable new insights into ancient Egyptian embalming
Source: Nature. 2023 Feb 1;614(7947):287–93. doi: 10.1038/s41586-022-05663-4 (PMC9908542; doi:10.1038/s41586-022-05663-4)
Supplement: Supplementary file 1 — The Supplementary Information contains Supplementary Sections 1 and 2 which present a detailed description of the archaeological context of the Saqqara complex (embalming workshop and ancient Egyptian embalming facilities) and of the results of the organic residue analyses (molecular assemblages associated with the identified substances). [file 41586_2022_5663_MOESM1_ESM.pdf]

nature portfolio

https://doi.org/10.1038/s41586-022-05663-4

Supplementary information

Biomolecular analyses enable new insights into ancient Egyptian embalming

In the format provided by the authors and unedited

Supplementary Information

Biomolecular analyses enable new insights into ancient Egyptian embalming

Maxime Rageot[1,2], Ramadan B. Hussein†[3], Susanne Beck[3], Victoria Altmann-Wendling[1,4], Mohammed I. M. Ibrahim[5], Mahmoud M. Bahgat[5], Ahmed M. Yousef[6], Katja Mittelstaedt[1], Jean-Jacques Filippi[7], Stephen Buckley[2,8], Cynthianne Spiteri[2,9], Philipp W. Stockhammer[1,10]

[1]Ludwig Maximilian University of Munich, Institute for Pre- and Protohistoric Archaeology and Archaeology of the Roman Provinces, Germany.

[2]Eberhard Karls University of Tübingen, Department of Pre- and Protohistory, Germany.

[3]Eberhard Karls University of Tübingen, Department of Egyptology, Germany

[4]Julius-Maximilians University, Würzburg, Department of Egyptology, Germany

[5] The Central Laboratories Network, the National Research Centre, Cairo, Egypt

[6] Packaging Materials Department, the National Research Centre, Cairo, Egypt

[7] Robertet S.A., Analytical research department, France

[8]University of York, BioArCh, United Kingdom

[9]University of Turin, Department of Life Sciences, Italy

[10]Max Planck Institute for Evolutionary Anthropology, Leipzig, Germany

Supplementary information 1 | Embalming workshop complex at Saqqara: new insights into the location and design of ancient Egyptian embalming facilities.

Embalming substances and embalming workshops are documented from three main sources: 1) archaeological deposits, conveniently known as "embalming caches"; 2) written and pictorial sources, e.g. embalming papyri and 3) organic residue analysis of Egyptian mummies. The embalming caches are associated with individual tombs and contain refuse of embalming materials including linen bandages, natron, pottery vessels, sawdust, and other detritus of the embalming procedure[1-8]. They are valuable material for interdisciplinary examinations, which cross the borders of the natural sciences and humanistic disciplines, as in, for example, biomolecular archaeology. The embalming papyri, described as embalming manuals, provide detailed accounts of the practical and ritual treatment of the dead body inside different facilities within the embalming workshop complex (e.g. the *per-nefer*, interpreted as "house of rejuvenation")[7,9-19]. The archaeological and textual sources reveal the great economic role of embalming[20-22]. Of significance are the *wabet*-facility (meaning "place/room of purification") and the *ibu*-purification tent/hall, where the main procedures of evisceration, body preservation, and wrapping were performed. During the mid-third millennium BCE, and based on their representations on tomb walls, both facilities were spatially separated and were possibly for individual use only. However, they were later, in the second millennium BCE, collapsed into the *per-nefer* workshop complexes, which were administered by entrepreneur priest-embalmers[3,12].

Our knowledge of the *per-nefer* (*pr-nfr*) embalming workshops has remained confined to pictorial and textual sources until the archaeologists of the University of Tübingen, Germany, working at Saqqara, uncovered the first embalming workshop complex of its kind. This unprecedented discovery, dating back to the 7th century BCE. (ca. 664–525 BCE), reshapes our knowledge as to the location and design of ancient Egyptian embalming facilities in the necropolis. Conjecture held that they were built aboveground, but the Saqqara workshop offers concrete evidence for embalming procedures having been carried out inside facilities both aboveground and underground. The embalming workshop is located a few meters to the south of the Pyramid of King Unas and includes a subterranean evisceration facility (Egyptian *wabet/wʿb.t*), a multifunctional aboveground structure (Egyptian *ibu/jb.w*), and communal burial spaces (i.e. Shaft K24, 30m deep, and catacomb-like galleries, Extended Data Fig. 6). The *wabet* room (8m × 5m × 3m) is located at the bottom of a 12.5m deep shaft and has an architectural design that could help reduce health risks for the ancient embalmers from microorganisms by means of: i) a ventilation system composed of an air tunnel running through the room's ceiling and connected to a massive network of other subterranean tunnels; ii) a simple two-channel drainage system for bodily fluids, cut on a ledge for evisceration (Extended Data Fig. 7); and iii) an industrial capacity fumigation source (i.e., a large pottery vessel, Extended Data Fig. 8) for repelling insects and deodorizing the room[23,24].

References

1 Budka, J. in *Archäologie und Ritual. Auf der Suche nach der rituellen Handlung in den antiken Kulturen Ägyptens und Griechenlands* (eds J. Mylonopoulos & H. Roeder) 85–103 (Phoibos-Verlag, 2006).

2 Aston, D. A. in *The Theban Necropolis. Past, Present and Future* (eds N. Strudwick & J. H. Taylor) 153–155 (The British Museum Press, 2003).

3 Chapman, S. L. *The Embalming Ritual of Late Period Through Ptolemaic Period Egypt* PhD thesis, Birmingham (2016).

4 Janák, J. & Landgráfová, R. in *Palaeopathology in Egypt and Nubia: a century in review* (eds R. Metcalfe, J. Cockitt, & R. David) 107-117 (Archaeopress, 2014).

5 Knoblauch, C. A new group of Middle Kingdom embalming deposits? Another look at pottery dumps and repositories for building materials in Middle Kingdom cemeteries. *Ägypten und Levante* **26**, 329-356, doi:10.1553/AEundL26. (2016).

6 Ikram, S. & López-Grande, M. J. Three embalming caches from Dra Abu el-Naga. *Bulletin de l'Institut Français d'Archéologie Orientale* **111**, 205-228 (2011).

7 Töpfer, S. *Das Balsamierungsritual. Eine (Neu-)Edition der Textkomposition Balsamierungsritual (pBoulaq 3, pLouvre 5158, pDurham 1983.11 + pSt. Petersburg 18128)*. (Harrassowitz. Studien zur spätägyptischen Religion 13., 2015).

8 Aston, D. A. & Aston, B. G. *Late period pottery from the New Kingdom necropolis at Saqqâra : Egypt Exploration Society--National Museum of Antiquities, Leiden, excavations 1975-1995*. (Egypt Exploration Society, 2010).

9 Donohue, V. A. in *Journal of Egyptian Archaeology* Vol. 64 43–148 (1978).

10 Frandsen, P. J. in *The Heritage of Ancient Egypt. Studies in Honor of Erik Iversen (Carsten Niebuhr Institute Publication)* (eds J. Osing & E. K. Nielsen) 49–62 (Museum Tusculanum Press, 1992).

11 Bardinet, T. *Médecins et magiciens à la cour du pharaon: une étude du papyrus médical Louvre E 32847*. (Khéops/musée du Louvre, 2018).

12 Vos, R. L. *The Apis embalming ritual: P. Vindob. 3873*. (Orientalia Lovaniensia Analecta 50, 1993).

13 Quack, J. F. in *Texte zur Wissenskultur* (eds B. Janowski & D. Schwemer) 418-438 (Gütersloh, 2020).

14 Möller, G. *Die beiden Totenpapyrus Rhind des Museums zu Edinburg*. (Hinrichs, 1913).

15 Recklinghausen, D. in *Ägyptische Mumien: Unsterblichkeit im Land der Pharaonen* (ed Anonymous) 53-61 (Philipp von Zabern, 2007).

16 Leitz, C. in *Ägyptische Mumien: Unsterblichkeit im Land der Pharaonen* (ed Anonymous) 167-177 (Philipp von Zabern, 2007).

17 Germer, R. *Mumien. Zeugen des Pharaonenreichs, Zurich/Munich*. (Artemis & Winkler, 1991).

18 Quack, J. F. *Zwei Handbücher der Mumifizierung im Balsamierungsritual des Apisstieres*. 123-129 (Enchoria 22, 1995).

19 Quack, J. F. *Beiträge zum Verständnis des Apisrituals*. 43-53 (Enchoria 24, 1997-1998).

20 Ikram, S. in *Apprivoiser le sauvage/Taming the Wild (CENiM 11)* (eds M. Massiera, B. Mathieu, & Fr. Rouffet) 211-228 (University Paul Valéry Montpellier 3, 2015).

21 Quack, J. F. in *Ägyptische Mumien. Unsterblichkeit im Land der Pharaonen* 19-27 (Landesmuseum Württemberg, 2007).

22 Cannata, M. in *Culture and History of the Ancient Near East 110* (Brill, 2020).

23 Hussein, R. B. & Marchand, S. A mummification workshop in Saqqara: the pottery from the main shaft K24. Saqqara Saite Tombs Project (SSTP). *Bulletin de liaison de la céramique égyptienne* **29**, 101-132 (2019).

24 Hussein, R. B. in *Guardian of Ancient Egypt. Studies in Honor of Zahi Hawass, II* (ed J. Kamrin et al.) 627–682 (Czech Institute of Egyptology, 2020).

Supplementary information 2 | Results of Organic Residue Analysis

Since the 35 vessels examined were discovered in two separate loci, they constitute two different groups. Nevertheless, each group stem from a single homogenous closed context. This indicates that the vessels were subject to the same taphonomic/natural alteration. Organic residues were identified in all vessels except for one, and lipid concentrations varied from 5 to 1653 μg per gram of sherd (Extended Data Table 1, Extended Data Fig. 1).

1. Conifer by-products: oils/tars of cedar and juniper/cypress

1.1. Cupressaceae by-product: oils/tars of juniper/cypress (n=21)

Totarols, and in some cases their derivatives (oxo-totarol), were identified in 21 samples (Fig. 2 and Extended Data Fig. 4). These phenolic diterpenoids are generally associated with the presence of members of the Cupressaceae family, in particular junipers or cypresses[25,26]. The absence of sandaracopimaric acid rules out the *Tetraclinis* (e.g., sandarac resin). Cuparene-related sesquiterpenes (especially cuparene and cuparenic acid) were identified in the 21 samples mentioned above (Fig. 2). They confirm the presence of juniper or cypress by-products[27-29]. Oxo-cuparene, an oxidised product of cuparene[28], was identified in seven vessels. Traces of α-muurolol (δ-cadinol) reported in juniper wood oil[27] and cypress oil[30] were also identified in five samples. In archaeological samples, the presence of low molecular weight sesquiterpenoids in quantities equal to or higher than that of the diterpenoids indicates that the substances were not resins but could have been prepared by steam-distillation / extractive immersion of odoriferous plant material (fragrance/essential oil[28,31,32]) or by pyrolysis (wood tar[33]).

In two additional samples (SSTP 33 and 104), cuparene-related sesquiterpenes were identified without totarol derivatives. They could also be associated with juniper/cypress oil as they are present in significant amounts in Cupressaceae. However, the identification of cuparenes alone is not always characteristic as they can be identified in different plants exudates/distillates[31,34,35] and cuparene may even be derived from the degradation of himachalenes[36] and therefore Cedrus sp.

1.2. Pinaceae by-product (n=20): oils/tars of cedar (n=19)

Abietane-type diterpenes (e.g., dehydroabietic and 7-oxo-dehydroabietic acids) were identified in 20 vessels. In four of these vessels isopimaric acid was also detected (SSTP 006, 020, 001, 104). This indicates the presence of conifer by-products (e.g. [37]), and Pinaceae by-products in archaeological contexts[25,26]. Sesquiterpenoid derivatives of the himachalene series were also identified in 19 of these vessels (Fig. 2 and Extended Data Fig. 4). γ-Dehydro-*ar*-himachalene, *ar*-himachalene and α-dehydro-*ar*-himachalene were detected in 17 vessels and only traces of the latter in two vessels. Traces of himachalol were also identified in nine vessels. The himachalenes are significant in cedar by-products[27,28,33,38-40] and can be considered as cedar oil

markers in archaeological contexts[28,41,42]. Oxidation products of *ar*-himachalenes, especially oxo-γ-dehydro-*ar*-himachalene and nor-*ar*-himachalone were also identified in 15 of the pots tested, along with oxo-*ar*-himachalol in three cases. These components attest to the intense oxidative alteration processes of the cedar by-product[28]. As for the Cupressaceae by-products, the predominance of sesquiterpenoids indicates the presence of an oil or tar.

Although, one or a few himachalenes have been detected in several plant extracts (e.g., *Abies sp.*[43], *Pimpinella sp.*[44], *Hypericum sp.* [45-47], *Juniperus sp.*[27,29,48,49]), these sesquiterpenes were to our knowledge 1/ never reported altogether in quantities large enough (always one or a few as traces / very low concentration in modern oil) to be relevant in the context of archaeological samples and 2/ rarely display the complete spectrum of himachalenes derivatives found in Cedrus SP (modern and artificially degraded[28,42]) and in the Saqqara samples. They can be considered as valid biomarkers because they are both 1/ originally present in Cedrus sp. by-products and 2/ formed by aromatisation/dehydrogenation of the native major sesquiterpenes of Cedrus sp., namely α-, β- and γ-himachalenes[42].

2. Angiosperm resins: elemi, pistacia resin and dammar

2.1. Burseraceae resin (n=16): Canarium resin (elemi)

The association of α- and β-amyrins and/or their derivatives strongly suggests the presence of a Burseraceae resin in 16 vessels, and their cooccurrence with lupeol in 12 vessels points to a Canarium resin also known as elemi[50-56] (Fig. 3 and Extended Data Table 2). Bursera and Protium resins can be excluded as they mainly occur in Central and South America[25,57]. 11-Keto amyrins (α and β) and/or their oxidized acetate derivatives were identified in 15 vessels (Extended Data Table 2). Among the different Burseraceae resins from Africa and Asia, these markers have been reported in Indian/Sri Lankan elemi (*Canarium zeylanicum*[50] and *Canarium strictum*[51,58]). Among the reference resins, which we have analysed under the same conditions as the archaeological samples (Fig. 3 and Extended Data Table 2), the same markers were identified in Asian elemis (*Canarium strictum* from India and *Canarium luzonicum* from the Philippines), and in 30-years-old frankincense (*Boswellia carterii/sacra*). This suite of markers, combined with the absence of epi-amyrins (α and β), epi-lupanes and boswellic acids/acetates, do not suggest a Boswellia resin (frankincense)[56,59-61] (Extended Data Table 2). Furthermore, 11-keto- α- and β-amyrin acetates were identified in 4 samples (SSTP 008, 030, 037 and 011), together with α- and β-amyrin acetates, 11-hydroxy-α-amyrin and 11-hydroxy-α-amyrin acetate in two of the sherds (SSTP 037 and 011; Fig. 3 and Extended Data Table 2). These markers occur naturally in the resin of *Canarium strictum*[51]. More specifically, traces of Brein (urs-12-ene-3,16-diol) were identified in two samples (SSTP 037 and 011). This biomarker was previously documented in Indonesian and Philippines elemi (e.g.[54,55]), as well as in our reference elemis from different origin (Asian and African) (Fig. 3 and Extended Data Table 2).

Finally, both olean-9(11),12-dien-3-ol and urs-9(11),12-dien-3-ol were detected in 14 samples (Extended Data Table 2), and both olean-9(11),12-dien-3-one and urs-9(11),12-dien-3-one in 3 sherds (SSTP 011, 019 and 001; Fig. 3). They have previously been identified as a result of

artificially aged elemis ("light" ageing) from Manila elemi and Mexican copal[53], and in some of the 30-years-old elemis investigated in this study.

2.2. Anacardiaceae resin (n=5): pistacia resin (mastic)

An assemblage of triterpenic markers from oleanane and tirucallane families, which include moronic, oleanonic, isomasticadienonic and masticadienonic acids, were identified in five pots (four as absorbed residues in ceramic vessels and one visible residue deposit in a red bowl from the burial chamber, Loc 4; Fig. 3). This distribution is characteristic of pistacia resin[26,32,56,62,63] (Fig. 3). In addition, 28-norolean-12,17-dien-3-one; 28-norolean-17-en-3-one were detected in two samples (SSTP 001 and 103), and possibly isomers of isomasticadienonic and masticadienonic acids (*m/z* 511) were identified in four samples (SSTP 001, 103, 008 and 020). These molecular constituents can be linked to a heat treatment[63].

2.3. Dipterocarpaceae resin (dammar; n=1)

An assemblage of triterpenic markers from dammarane, nor-ursane and oleanane families was identified in the organic extract absorbed in the ceramic fabric of a goldfish bowl from the burial chamber, Loc. 4 (SSTP 001b; Fig. 3 and Extended Data Fig. 1). These include dammaradien-3-ol, nor-α-amyrone and δ-amyrone (olean-13(18)-en-3-one), which were previously identified in modern and archaeological Dipterocarpaceae resin[52,62,64-67], as well as in the 30-years-old dammar resin (*Shorea selamica*) which was analysed under the same conditions as the archaeological sample (Fig. 3). To the best of our knowledge, these three biomarkers have never been identified together in any other resin. Except for the identification of traces of dammaradien-3-ol in a fresh resin of *pistacia terebinthus*[68], we did not found any mention in the literature of their identification in resins of elemi, Commiphora, Boswelia and pistacia[50-56,62,68-75]. Samples of modern/30-years-old resins investigated in our study under the same conditions as the archaeological samples provided the same evidence (elemis, Commiphora, Boswelia and pistacia resins).

We have also identified an additional marker of dammar which is the 20,24-epoxy-25-hydroxydammaren-3-ol (product of the oxidation of hydroxydammarenol[73]). Although derivatives of this compound have been identified in modern extract of *Commiphora sp*[69,71], the amount of dammaranes reported by these authors precludes their relevance as markers of *Commiphora sp.* in an archaeological context. Therefore, 20,24-epoxy-25-hydroxydammaren-3-ol can reasonably be used as another argument for the presence of dammar in our samples.

In addition to this molecular assemblage characteristic of dammar, further markers of dammar resin were identified. However, these are less specific because they are also detected in other resins, especially from *Pistacia*. In particular, Hexakisnor-dammarandione is reported in modern and aged dammar resin[52] (Fig. 3) and in archaeological pistacia resin[63,76]; nor-β-amyrone, oleanonic aldehyde and dammaradien-3-one are documented in modern, aged and archaeological dammar resin[52,62,64-66,72,73,77] (Fig. 3) and pistacia resins[32,56,62,68,73,75] (Fig. 3). In this Saqqara sample, these compounds can be linked to the dammar components, as the characteristic and main triterpenic biomarkers of pistacia resin (moronic, oleanolic, isomasticadienonic and masticadienonic acids) were not detected. Amyrin derivatives (α-

amyrin and β/α-amyrone) were also identified in this sample, and have been documented in dammar resin[64,66] (Fig. 3) as well as in several Burseraceae resins[50,51,53,60]. Finally, degraded markers which can result from natural degradation and/or heating processes of oleananes, were identified (Fig. 3). These were previously reported in modern and archaeological dammar (Olean-9(11),12-dien-3-one[62,64,66]) and in archaeological pistacia resin (olean-9(11) en-3-one; olean-18-en-3-one; olean-18-en-3-one; 28-norolean-12, 17-dien-3-one[32,63]).

3. Animal fats, plant oil and beeswax

Fatty acids, especially palmitic and stearic acids were identified in 30 vessels, which may suggest the presence of animal fat, plant oil and/or beeswax according to their distribution.

3.1. Animal fats (n=18)

A narrow distribution of saturated triacylglycerols (TAGs) was identified in 13 vessels and shows the presence of animal fats (e.g.[78]). TAGs 46:0 or 48:0 to 54:0 are identified in 10 vessels (SSTP 001, 103, 031, 017, 037, 030, 038, 016, 039, 040, Extended Data Fig. 2). TAGs with up to 52:0 were present in one sample (SSTP 014), and only TAGs 48:0 and 50:0 were identified in two others (SSTP 008 and 018). A distribution of saturated DAGs (36:0, 34:0 and 32:0 isomers) resulting from the first step of degradation of the TAGs was also identified in all of these samples, and in 6 additional vessels (SSTP 006, 024, 005, 007, 032). This shows the presence of animal fat in 18 vessels (Extended Data Fig. 1). Furthermore, traces of saturated TAGs with an odd number of carbon atoms (53:0, 51:0 and 49:0) were identified in 7 vessels (SSTP 016, 038, 037, 039, 040; only TAGs 51 for SSTP 001 and TAGs 47 in STPP 030; Extended Data Fig. 2 and Extended Data Fig. 1). These are characteristic of ruminant animal fats[32,79]. The major saturated fatty acids present in animal fat, namely palmitic, stearic and myristic acids (e.g.[80]), were identified in all 18 samples and in 8 additional ones for which the animal or plant origin could not be clearly determined (Extended Data Fig. 1). A mixture of positional isomers of octadecenoic acid ($C_{18:1}$) and minor straight and branched pentadecanoic and heptadecanoic acids was also identified in the 7 samples associated with the odd-numbered TAGs, as well as in 9 additional samples (SSTPP 006, 023, 024, 005, 007, 008, 031, 036 and 103; Extended Data Fig. 1 and 4). Minor straight and branched pentadecanoic and heptadecanoic acids are known to be formed in the rumen by bacterial synthesis, and thus might be assigned to ruminant animal fats (e.g.[78]). The formation of several positional $C_{18:1}$ can result from the biohydrogenation of dietary fats which occurs in the rumen of ruminant animals[81].

3.2. Plant oil (n=5)

Unsaturated triacylglycerols (54:3, 52:2 and 50:1) were detected in 4 samples (SSTP 010, 037, 014, 031). Unsaturated diacylglycerols (DAGs 34:1 and 36:2 isomers) and di-palmitin (DAGs 32:0 isomers) were also identified (Extended Data Fig. 3). They result from the degradation of the unsaturated TAGs. Unsaturated TAGs and DAGs are extremely scarce in archaeological residues, as they are rarely preserved. Only plants that contain high quantities of oils will lead to the preservation of unsaturated TAGs. Despite their very poor preservation in most of

archaeological contexts, these markers are almost the only ones that can be used to identify the nature of archaeological plant oils[79,82]. The distribution of TAGs in SSTP 010, 037 and 014 corresponds to modern olive oil. However, other oils containing the same TAGs co-occurring with other even more easily degraded polyunsaturated TAGs (e.g., argan and hazelnut oil) cannot be totally excluded[79,82]. In a fifth pot (SSTP 020), ricinoleic acid was identified (Extended Data Fig. 4). This marker suggests the presence of castor oil in ancient Egyptian contexts[80,83]. However, it could also derive from lipids biosynthesized by the ergot fungi which are common pests of Graminea, and therefore also could be associated with the presence of cereal[84]. In the Saqqara embalming workshop, the presence of castor oil (in mixture with cedar oil, Pistacia resin, elemi and possibly mixed with other oils) is most plausible as the vessels are dedicated to the preparation of balsams for mummification, which required antiseptic/antifungal properties. Palmitic and oleic acids, identified in the five samples, were the major fatty acids in SSTP 010, 014 and 020. Traces of linoleic acid were also identified in SSTP 010 and 014. Azelaic acid and the two isomers of 9,11-dihydroxy-stearic acids were also identified in SSTP 020 as well as in SSTP 031 and SSTP 037. The formation of the hydroxy acids can result from oxidation by dihydroxylation of the double bond in oleic acid[85]. The presence of 9- and 10-hydroxyoctadecenoic acids can result from the dehydration of the two isomers[85].

3.3. Beeswax (n=5)

Long-chain palmitic esters with an even number of carbon atoms from C40 to C48 and saturated long-chain even-numbered fatty acids ($C_{22:0}$ to $C_{28:0}$, maximising at C24:0) characteristic of beeswax[86,87] (Fig. 3) were identified in 5 vessels Extended Data Fig. 1). The presence of palmitic acid in each, and that of even-numbered *n*-alcohols with 22 to 30 carbon atoms in 3 samples (SSTP 39, 43 and 103) can be attributed to a process of preferential hydrolysis of shorter-chain esters[87,88]. The series of odd-number *n*-alkanes which are indicative of beeswax were not identified in the five vessels. This suggests that the bee-products were subjected to a heat treatment[87].

4. Fossil organic product (bitumen)

The presence of characteristic hopanes, i.e., 17α(H), 22,29,30-trisnorhopane; 17α(H),21β(H)-norhopane; 17 α(H),21β(H)-hopane; homohopanes; bishomohopane, trishomohopanes; tetrakishomohopanes; pentakishomohopanes and steranes, i.e. cholestanes, ergostanes, stigmastanes in 2 pottery samples (SSTP 001 and104), indicate the presence of bitumen[41,89-94] (Extended Data Fig. 5). It is not totally excluded that bitumen is under-represented if it is in a lower proportion than in both samples. Indeed, the small amount of material absorbed in the ceramics limited the application of targeted methods for bitumen detection[34,35].

The presence of gammacerane and the absence of oleanane, 27 diasteranes and norneohopanes can suggest Dead Sea bitumen rather than a more local source in the Gulf of Suez, such as Abu Durba (Sinai) or Gebel Zeit[35,41,92]. The hopane C35 index (= 17α (H),21β(H)-29-pentakishomohopanes / (17α (H),21β(H)-29-pentakishomohopanes + 17α (H),21β(H)-29-

trishomohopanes)[95], which was calculated for both samples (SSTP 104= 0,54 and SSTP 001= 0,62) is indicative of a Dead see bitumen[41]. In the bitumen found at Saqqara, the concentration of C27, C28 and C29 steranes are equal, similarly to the Dead Sea bitumen and in contrast to the source at Hit[92].

5. Markers of concoctions/mixtures of bio-products

Triterpenic palmitates were identified in 3 vessels. They can result from the intentional mixing of triterpenic tar/resins and fats[96]. 11-Oxo-β-amyrin palmitate, 11-oxo-α-amyrin palmitate and α-amyrin palmitate were characterised in 2 white beakers (SSTP 011 and SSTP 037). Their identification in association with the other markers of elemi, animal fat and/or plant oil indicates an intentional preparation involving the mixing and heating of these substances (Fig. 3 and Extended Data Fig. 1). Another recipe based on dammar resin, beeswax and/or animal fat markers can be proposed based on the presence of their specific markers as well as α-amyrin palmitate and oxo-oleanene palmitate in a red bowl (SSTP 001; Fig. 3 and Extended Data Fig. 1).

References

25 Mills, J. S. & white, R. Natural resins of art and archaeology their sources, chemistry, and identification. *Studies in Conservation* **22**, 12-31, doi:doi:10.1179/sic.1977.003 (1977).

26 Mills, J. S. & White, R. *The organic chemistry of museum objects*. second edn, (Butterworth-Heineman, 1994).

27 Saab, A. M. *et al.* Phytochemical analysis and cytotoxicity towards multidrug-resistant leukemia cells of essential oils derived from Lebanese medicinal plants. *Planta Med* **78**, 1927-1931, doi:10.1055/s-0032-1327896 (2012).

28 Sarret, M. *et al.* Organic substances from Egyptian jars of the Early Dynastic period (3100–2700BCE): Mode of preparation, alteration processes and botanical (re)assessment of "cedrium". *Journal of Archaeological Science: Reports* **14**, 420-431, doi:https://doi.org/10.1016/j.jasrep.2017.06.021 (2017).

29 Chalchat, J. C., Ph. Garry, R. & Michet, A. Chemical composition of the hexane fraction of empyreumatic wood oils from juniperus oxycedrus L. and Juniperus phoenicea L. *Flavour and Fragrance Journal* **3**, 19-22, doi:10.1002/ffj.2730030104 (1988).

30 Kuiate, J.-R., Bessière, J. M., Zollo, P. H. A. & Kuate, S. P. Chemical composition and antidermatophytic properties of volatile fractions of hexanic extract from leaves of Cupressus lusitanica Mill. from Cameroon. *Journal of Ethnopharmacology* **103**, 160-165, doi:https://doi.org/10.1016/j.jep.2005.07.022 (2006).

31 Vandenbeusch, M., Stacey, R. & Antoine, D. Rediscovering Nestawedjat: Embalming residue analyses reunite the mummified remains of an ancient Egyptian woman with her coffins. *Journal of Archaeological Science: Reports* **40**, 103186, doi:https://doi.org/10.1016/j.jasrep.2021.103186 (2021).

32 Charrié-Duhaut, A. *et al.* The canopic jars of Rameses II: real use revealed by molecular study of organic residues. *Journal of Archaeological Science* **34**, 957-967, doi:http://dx.doi.org/10.1016/j.jas.2006.09.012 (2007).

33 Rageot, M. *Les substances naturelles en Méditerranée nord-occidentale (VIe-Ie Millénaire BCE) : chimie et archéologie des matériaux exploités pour leurs propriétés adhésives et hydrophobes* Thése de doctorat thesis, Université Nice Sophia Antipolis, (2015).

34 Łucejko, J., Connan, J., Orsini, S., Ribechini, E. & Modugno, F. Chemical analyses of Egyptian mummification balms and organic residues from storage jars dated from the Old Kingdom to the Copto-Byzantine period. *Journal of Archaeological Science* **85**, 1-12, doi:https://doi.org/10.1016/j.jas.2017.06.015 (2017).
35 Fulcher, K., Serpico, M., Taylor, J. H. & Stacey, R. Molecular analysis of black coatings and anointing fluids from ancient Egyptian coffins, mummy cases, and funerary objects. *Proceedings of the National Academy of Sciences* **118**, e2100885118, doi:doi:10.1073/pnas.2100885118 (2021).
36 Subba Rao, H. N., Damodaran, N. P. & Dev, S. A one-step conversion of (+)-β-himachalene into (+)-cuparene. *Tetrahedron Letters* **9**, 2213-2214, doi:https://doi.org/10.1016/S0040-4039(00)89722-1 (1968).
37 van den Berg, K. J., Boon, J. J., Pastorova, I. & Spetter, L. F. M. Mass spectrometric methodology for the analysis of highly oxidized diterpenoid acids in Old Master paintings. *Journal of Mass Spectrometry* **35**, 512-533, doi:10.1002/(sici)1096-9888(200004)35:4<512::aid-jms963>3.0.co;2-3 (2000).
38 Başer, K. H. C. & Demirçakmak, B. The essential oil of taurus cedar (Cedrus libani A. rich): Recent results. *Chemistry of Natural Compounds* **31**, 16-20, doi:10.1007/BF01167563 (1995).
39 Aberchane, M., Fechtal, M. & Chaouch, A. Analysis of Moroccan Atlas cedarwood oil (Cedrus atlantica Manetti). *Journal of essential oil research : JEOR.* **16**, 542-547, doi:10.1080/10412905.2004.9698793 (2004).
40 Loizzo, M. R., Saab, A. M., Statti, G. A. & Menichini, F. Composition and α-amylase inhibitory effect of essential oils from Cedrus libani. *Fitoterapia* **78**, 323-326, doi:https://doi.org/10.1016/j.fitote.2007.03.006 (2007).
41 Buckley, S., clark, K. A. & Evershed, R. P. Complex organic chemical balms of Pharaonic animal mummies. *Nature* **431**, 294-299 (2004).
42 Huber, B., Vassão, D. G., Roberts, P., Wang, Y. V. & Larsen, T. Chemical Modification of Biomarkers through Accelerated Degradation: Implications for Ancient Plant Identification in Archaeo-Organic Residues. *Molecules* **27**, doi:10.3390/molecules27103331 (2022).
43 Bagci, E., Başer, K. H. C., Kurkcuoglu, M., Babaç, T. & Çelik, S. Study of the essential oil composition of two subspecies of Abies cilicica (Ant. et Kotschy) Carr. from Turkey. *Flavour and Fragrance Journal* **14**, 47-49 (1999).
44 Tabanca, N. *et al.* Gas chromatographic-mass spectrometric analysis of essential oils from Pimpinella species gathered from Central and Northern Turkey. *Journal of chromatography. A* **1117**, 194-205, doi:10.1016/j.chroma.2006.03.075 (2006).
45 Petrakis, P. V., Couladis, M. & Roussis, V. A method for detecting the biosystematic significance of the essential oil composition: The case of five Hellenic Hypericum L. species. *Biochemical Systematics and Ecology* **33**, 873-898, doi:https://doi.org/10.1016/j.bse.2005.02.002 (2005).
46 Schwob, I., Bessière, J. M. & Viano, J. Composition of the essential oils of Hypericum perforatum L. from southeastern France. *Comptes rendus biologies* **325**, 781-785, doi:10.1016/s1631-0691(02)01489-0 (2002).
47 Maggi, F. & Ferretti, G. Essential Oil Comparison of Hypericum perforatum L. subsp. perforatum and subsp. veronense (Schrank) Ces. from Central Italy. *Journal of Essential Oil Research* **20**, 492 - 494 (2008).
48 Gonny, M., Cavaleiro, C., Salgueiro, L. & Casanova, J. Analysis of Juniperus communis subsp. alpina needle, berry, wood and root oils by combination of GC, GC/MS and 13C-NMR. *Flavour and Fragrance Journal* **21**, 99-106, doi:10.1002/ffj.1527 (2006).
49 el-Sawi, S. A., Motawae, H. M. & Ali, A. M. Chemical composition, cytotoxic activity and antimicrobial activity of essential oils of leaves and berries of Juniperus phoenicea L. grown in Egypt. *African journal of traditional, complementary, and alternative medicines : AJTCAM* **4**, 417-426, doi:10.4314/ajtcam.v4i4.31236 (2007).
50 Bandaranayake, W. M. Terpenoids of Canarium zeylanicum. *Phytochemistry* **19**, 255-257, doi:https://doi.org/10.1016/S0031-9422(00)81969-X (1980).

51 Hinge, V. K., Wagh, A. D., Paknikar, S. K. & Bhattacharyya, S. C. Terpenoids—LXXI: Constituents of Indian black dammar resin. *Tetrahedron* **21**, 3197-3203, doi:https://doi.org/10.1016/S0040-4020(01)96937-6 (1965).

52 Cartoni, G., Russo, M. V., Spinelli, F. & Talarico, F. GC-MS Characterisation and Identification of Natural Terpenic Resins Employed in Works of Art. *Annali di Chimica* **94**, 767-782, doi:10.1002/adic.200490098 (2004).

53 De la Cruz-Cañizares, J., Doménech-Carbó, M.-T., Gimeno-Adelantado, J.-V., Mateo-Castro, R. & Bosch-Reig, F. Study of Burseraceae resins used in binding media and varnishes from artworks by gas chromatography–mass spectrometry and pyrolysis-gas chromatography–mass spectrometry. *Journal of Chromatography A* **1093**, 177-194, doi:https://doi.org/10.1016/j.chroma.2005.07.058 (2005).

54 Kikuchi, T. *et al.* Melanogenesis Inhibitory Activity of Sesquiterpenes from Canarium ovatum Resin in Mouse B16 Melanoma Cells. *Chemistry & Biodiversity* **9**, 1500-1507, doi:10.1002/cbdv.201200111 (2012).

55 Steigenberger, G. *The Vigani Cabinet – Analysis of historical resinous materials by gas chromatography - mass spectrometry and infrared spectroscopy* PhD thesis, Technical University Dresden, (2013).

56 Bruni, S. & Guglielmi, V. Identification of archaeological triterpenic resins by the non-separative techniques FTIR and 13C NMR: The case of Pistacia resin (mastic) in comparison with frankincense. *Spectrochimica Acta Part A: Molecular and Biomolecular Spectroscopy* **121**, 613-622, doi:http://dx.doi.org/10.1016/j.saa.2013.10.098 (2014).

57 Langenheim, J. H. *Plant Resines : chemistry, evolution, ecology, and ethnobotany*. (Timber Press, 2003).

58 Hinge, V. K., Paknikar, S. K., Dast, K. G., Bose, A. K. & Bhattacharyya, S. C. Terpenoids—LXXXVI. Structure of epi-ψ-taraxastanonol and epi-ψ-taraxastanedio. *Tetrahedron* **22**, 2861-2868 (1966).

59 Evershed, R. P. *et al.* Archaeological frankincense. *Nature* **390**, 667-668, doi:10.1038/37741 (1997).

60 Mathe, C., Culioli, G., Archier, P. & Vieillescazes, C. Characterization of archaeological frankincense by gas chromatography–mass spectrometry. *Journal of Chromatography A* **1023**, 277-285, doi:http://dx.doi.org/10.1016/j.chroma.2003.10.016 (2004).

61 Regert, M., Devièse, T., Le Hô, A.-S. & Rougeulle, A. Reconstructing ancient Yemeni commercial routes during the middle Ages using structural characterization of terpenoid resins. *Archaeometry* **50**, 668-695 (2008).

62 van der Doelen, G. A. *et al.* Analysis of fresh triterpenoid resins and aged triterpenoid varnishes by high-performance liquid chromatography–atmospheric pressure chemical ionisation (tandem) mass spectrometry. *Journal of Chromatography A* **809**, 21-37, doi:https://doi.org/10.1016/S0021-9673(98)00186-1 (1998).

63 Stern, B., Heron, C., Corr, L., Serpico, M. & Bourriau, J. Compositional variations in aged and heated pistacia resin found un Late Bronze age Canaanite amphorae and bowls from Arman, Egypt. *Archaeometry* **45**, 457–469 (2003).

64 Burger, P., Charrié-Duhaut, A., Connan, J., Flecker, M. & Albrecht, P. Archaeological resinous samples from Asian wrecks: Taxonomic characterization by GC–MS. *Analytica Chimica Acta* **648**, 85-97 (2009).

65 Burger, P. *CARACTERISATION MOLECULAIRE DE RESINES VEGETALES ARCHEOLOGIQUES ET ACTUELLES : ETUDE DE RESINES DE DIPTEROCARPACEAE*, Université de Strasbourg, (2008).

66 Charrié-Duhaut, A., Burger, P., Maurer, J., Connan, J. & Albrecht, P. Molecular and isotopic archaeology: Top grade tools to investigate organic archaeological materials. *Comptes Rendus Chimie* **12**, 1140-1153 (2009).

67 Colombini, M. P. & Modugno, F. *Organic Mass Spectrometry in Art and Archaeology*. (Willey, 2009).

68 Assimopoulou, A. N. & Papageorgiou, V. P. GC-MS analysis of penta- and tetra-cyclic triterpenes from resins of Pistacia species. Part I. Pistacia lentiscus var. Chia. *Biomedical Chromatography* **19**, 285-311, doi:10.1002/bmc.454 (2005).
69 Dekebo, A., Dagne, E., Curry, P., Gautun, O. R. & Aasen, A. J. Dammarane triterpenes from the resins of Commiphora confusa. *Bulletin of the Chemical Society of Ethiopia* **16**, 81-86 (2002).
70 Dekebo, A., Dagne, E., Gautun, O. R. & Aasen, A. J. Triterpenes from the resin of Boswellia neglecta. *Bulletin of The Chemical Society of Ethiopia* **16**, 87-90 (2002).
71 Waterman, P. G. & Ampofo, S. Dammarane Triterpenes from the stem bark of Commiphora Dalzielii. *Phytochemistry* **24**, 2925-2928, doi:https://doi.org/10.1016/0031-9422(85)80029-7 (1985).
72 van der Doelen, G. A., van den Berg, K. J. & Boon, J. J. Comparative chromatographic and mass-spectrometric studies of triterpenoid varnishes: fresh material and aged samples from paintings. *Studies in Conservation* **43**, 249-264, doi:10.1179/sic.1998.43.4.249 (1998).
73 van der Doelen, G. A. & Boon, J. J. Artificial ageing of varnish triterpenoids in solution. *Journal of Photochemistry and Photobiology A: Chemistry* **134**, 45-57, doi:https://doi.org/10.1016/S1010-6030(00)00245-8 (2000).
74 Colombini, M. P., Modugno, F., Giannarelli, S., Fuoco, R. & Matteini, M. GC-MS characterization of paint varnishes. *Microchemical Journal* **67**, 385-396, doi:https://doi.org/10.1016/S0026-265X(00)00098-9 (2000).
75 Assimopoulou, A. N. & Papageorgiou, V. P. GC-MS analysis of penta- and tetra-cyclic triterpenes from resins of Pistacia species. Part II. Pistacia terebinthus var. Chia. *Biomedical chromatography : BMC* **19**, 586-605, doi:10.1002/bmc.484 (2005).
76 Koller, J., Baumer, U., Kaup, Y. & Weser, U. Herodotus' and Pliny's Embalming Materials Identified on Ancient Egyptian Mummies. *Archaeometry* **47**, 609–628 (2005).
77 Dutta, S., Saxena, R. & Singh, H. Exceptional preservation of angiosperm markers in Miocene and Eocene ambers. *Geology* **42**, doi:10.1130/G34975.1 (2014).
78 Regert, M. Analytical strategies for discriminating archeological fatty substances from animal origin. *Mass Spectrometry Reviews* **30**, 177-220 (2011).
79 Garnier, N., Rolando, C., Høtje, J. M. & Tokarski, C. Analysis of archaeological triacylglycerols by high resolution nanoESI, FT-ICR MS and IRMPD MS/MS: Application to 5th century BC–4th century AD oil lamps from Olbia (Ukraine). *International Journal of Mass Spectrometry* **284**, 47-56, doi:http://dx.doi.org/10.1016/j.ijms.2009.03.003 (2009).
80 Yatsishina, E. B., Pozhidaev, V. M., Sergeeva, Y. E., Malakhov, S. N. & Slushnaya, I. S. An Integrated Study of the Hair Coating of Ancient Egyptian Mummies. *Journal of Analytical Chemistry* **75**, 262-274, doi:10.1134/S1061934819120141 (2020).
81 Evershed, R. P. *et al.* New Criteria for the Identification of Animal Fats Preserved in Archaeological Pottery. *Naturwissenschaften* **84**, 402-406, doi:10.1007/s001140050417 (1997).
82 Rageot, M. *et al.* New insights into Early Celtic consumption practices: Organic residue analyses of local and imported pottery from Vix-Mont Lassois. *PLoS ONE* **14** (2019).
83 Copley, M. S., Bland, H. A., Rose, P., Horton, M. & Evershed, R. P. Gas chromatographic, mass spectrometric and stable carbon isotopic investigations of organic residues of plant oils and animal fats employed as illuminants in archaeological lamps from Egypt. *Analyst* **130**, 860-871, doi:10.1039/B500403A (2005).
84 Lucejko, J. J. *et al.* Long-lasting ergot lipids as new biomarkers for assessing the presence of cereals and cereal products in archaeological vessels. *Scientific Reports* **8**, 3935, doi:10.1038/s41598-018-22140-z (2018).
85 Regert, M., Bland, H. A., Dudd, S. N., Bergen, P. F. v. & Evershed, R. P. Free and bound fatty acid oxidation products in archaeological ceramic vessels. *Proc. R. Soc. Lond. B* **265**, 2027-2032 (1998).
86 Evershed, R. P., Vaughan, S. J., Dudd, S. N. & Soles, J. S. Fuel for thought? Beeswax in lamps and conical cups from Late Minoan Crete. *Antiquity* **71**, 979-985 (1997).

87 Regert, M., Colinart, S., Degrand, L. & Decavallas, O. Chemical alteration and use of beeswax through time : accelerated ageing test and analysis of archaeological samples from various environmental contexts. *Archaeometry* **43**, 549-569 (2001).
88 Lattuati-Derieux, A., Thao, S., Langlois, J. & Regert, M. First results on headspace-solid phase microextraction-gas chromatography/mass spectrometry of volatile organic compounds emitted by wax objects in museums. *Journal of Chromatography A* **1187**, 239-249 (2008).
89 Connan, J. & Deschesne, O. Archaeological bitumen: Identification, origins and uses of an ancient Near easter material. *Materials Research Society Symposium Proceedings* **267**, 683-720 (1992).
90 Connan, J. & Ourisson, G. De la géochimie pétrolière à l'étude des bitumes anciens : l'archéologie moléculaire. *Comptes rendus des séances de l'Académie des Inscriptions et Belles-Lettres*, 901-921 (1993).
91 Connan, J. Use and trade of bitumen in antiquity and prehistory: molecular archaeology reveals secrets of past civilizations. *Philos Trans R Soc Lond B Biol Sci.* **354** 33–50 (1999).
92 Clark, K. A. *Tracing the Evolution of Organic Balm used in Egyptian Mummification via Molecular and Isotopic Signatures*, University of Bristol, (2006).
93 Clark, K. A., Ikram, S. & Evershed, R. P. The significance of petroleum bitumen in ancient Egyptian mummies. *Philos Trans A Math Phys Eng Sci* **374**, 20160229, doi:10.1098/rsta.2016.0229 (2016).
94 Fulcher, K. & Budka, J. Pigments, incense, and bitumen from the New Kingdom town and cemetery on Sai Island in Nubia. *Journal of Archaeological Science: Reports* **33**, 102550, doi:https://doi.org/10.1016/j.jasrep.2020.102550 (2020).
95 Harrell, J. A. & Lewan, M. D. Sources of mummy bitumen in ancient Egypt and Palestine. *Archaeometry* **44**, 285-293 (2002).
96 Dudd, S. N. & Evershed, R. P. Unsual triterpenoid fatty acyl ester component of archaeological birch bark tars. *Tetrahedron Letters* **40**, 359-362 (1999).
