# Supplementary material for: Biomolecular analyses enable new insights into ancient Egyptian embalming
Source: Nature. 2023 Feb 1;614(7947):287–93. doi: 10.1038/s41586-022-05663-4 (PMC9908542; doi:10.1038/s41586-022-05663-4)
Supplement: Supplementary file 2 — Reporting Summary [file 41586_2022_5663_MOESM2_ESM.pdf]

nature portfolio

Corresponding author(s): Maxime Rageot, Philipp W. Stockhammer

Last updated by author(s): Nov 25, 2022

Reporting Summary

Nature Portfolio wishes to improve the reproducibility of the work that we publish. This form provides structure for consistency and transparency in reporting. For further information on Nature Portfolio policies, see our Editorial Policies and the Editorial Policy Checklist.

Statistics

For all statistical analyses, confirm that the following items are present in the figure legend, table legend, main text, or Methods section.

n/a	Confirmed	
☒	☐	The exact sample size (n) for each experimental group/condition, given as a discrete number and unit of measurement
☒	☐	A statement on whether measurements were taken from distinct samples or whether the same sample was measured repeatedly
☒	☐	The statistical test(s) used AND whether they are one- or two-sided<br>*Only common tests should be described solely by name; describe more complex techniques in the Methods section.*
☒	☐	A description of all covariates tested
☒	☐	A description of any assumptions or corrections, such as tests of normality and adjustment for multiple comparisons
☒	☐	A full description of the statistical parameters including central tendency (e.g. means) or other basic estimates (e.g. regression coefficient) AND variation (e.g. standard deviation) or associated estimates of uncertainty (e.g. confidence intervals)
☒	☐	For null hypothesis testing, the test statistic (e.g. F, t, r) with confidence intervals, effect sizes, degrees of freedom and P value noted<br>*Give P values as exact values whenever suitable.*
☒	☐	For Bayesian analysis, information on the choice of priors and Markov chain Monte Carlo settings
☒	☐	For hierarchical and complex designs, identification of the appropriate level for tests and full reporting of outcomes
☒	☐	Estimates of effect sizes (e.g. Cohen's d, Pearson's r), indicating how they were calculated

Our web collection on statistics for biologists contains articles on many of the points above.

Software and code

Policy information about availability of computer code

Data collection	No software was used for data collection
Data analysis	ChemStation for GC and GC-MS software (Agilent) was used to collect the data analysed by GC/GC-MS

For manuscripts utilizing custom algorithms or software that are central to the research but not yet described in published literature, software must be made available to editors and reviewers. We strongly encourage code deposition in a community repository (e.g. GitHub). See the Nature Portfolio guidelines for submitting code & software for further information.

Data

Policy information about availability of data

All manuscripts must include a data availability statement. This statement should provide the following information, where applicable:
- Accession codes, unique identifiers, or web links for publicly available datasets
- A description of any restrictions on data availability
- For clinical datasets or third party data, please ensure that the statement adheres to our policy

All information on the samples and the data generated and analysed in this study are included in the manuscript, supplementary information files and Extended Data files.

Field-specific reporting

Please select the one below that is the best fit for your research. If you are not sure, read the appropriate sections before making your selection.

☐ Life sciences ☒ Behavioural & social sciences ☐ Ecological, evolutionary & environmental sciences

For a reference copy of the document with all sections, see nature.com/documents/nr-reporting-summary-flat.pdf

Behavioural & social sciences study design

All studies must disclose on these points even when the disclosure is negative.

Study description	Integrating archaeological, philological, and organic residue analyses, we shed a new light on the practice and economy of embalming in ancient Egypt. The organic contents of 31 ceramic vessels recovered from a 26th Dynasty embalming workshop at Saqqara were analysed as well as four samples from two burial chambers (qualitative method). These vessels are labelled according to their content and/or use, thus enabling us to correlate organic substances with both Egyptian names and particular embalming practices. Specific mixtures were identified of fragrant/antiseptic oils, tars, and resins that were used for embalming the head and treating the wrappings. The identification of non-local organic substances allows the reconstruction of trade networks that provided ancient Egyptian embalmers with the substances required for mummification. This extensive demand for foreign products fostered trade both within the Mediterranean (e.g., Pistacia and conifer by-products) and with tropical forest regions (dammar, elemi). Additionally, we show that at Saqqara the substances of "antiu" and "sefet"—well-known from ancient texts and usually translated as "myrrh/incense" and "a sacred oil"—should be respectively understood as a coniferous oils/tars -based mixture and an unguent with plant additives.
Research sample	A German-Egyptian academic collaboration was built and enabled to jointly conduct high-end organic residue analysis at the National Research Centre of Egypt and establish the first laboratory for organic residue analysis in Egypt. In this laboratory, we were now able to study 31 vessels found during the recent excavation of an embalmers' workshop and four samples from two burial chambers at Saqqara, dating to the early 1st millennium BCE. The vessels from this workshop are labelled with the substances used during embalming and/or the instruction where or how to apply the respective substance.
Sampling strategy	The selection of archaeological samples followed a list of criteria which took into consideration the archaeological context, secure stratigraphic contexts, and the overall vessel shape (red goldfish bowls and whit marl clay beakers) and readability of embalming-related labels. We selected nine beakers and 22 red bowls with the clearest readable labels for ORA from the embalming workshop as well as four samples (two red bowls, one faience cup, and one red cylindrical vessel) from two burial chambers (Loc. 3 and Loc. 4) located at the bottom of the communal burial shaft .<br>One gram of potsherd was drilled (Layer 2) following cleaning of the vessel surfaces to remove any exogenous lipids (Layer 1). This is the commonly accepted amount needed to solvent extract the organic residues impregnated in the ceramics.<br>The characterization of the lipid constituents present (using GC-MS) was based on the analytical results obtained from Layer 2. The ceramic powder collected during surface cleaning (Layer 1) was retained for potential additional analysis.
Data collection	At the site of Saqqara (Deposit room-magazine), one gram of each 35 potsherds was drilled (Layer 2) following cleaning of the vessel surfaces to remove any exogenous lipids. Organic residue analysis (Organic residue extraction, sample preparation and GC-MS analyses) was carried out at the National Research Centre (NRC), Chromatographic Laboratories Network, Giza, Egypt. All information related to the samples was recorded in an Excel file (table in Supplementary Information). The results of the GC-MS analyses were read using the Chemstation software from Agilent.
Timing	Data collection (sampling) took place in November 2019 and Organic residue analysis in November 2019 and February 2020.
Data exclusions	No Data were excluded from the analysis
Non-participation	No participants dropped out / declined participation.
Randomization	*If participants were not allocated into experimental groups, state so OR describe how participants were allocated to groups, and if allocation was not random, describe how covariates were controlled.*

Reporting for specific materials, systems and methods

We require information from authors about some types of materials, experimental systems and methods used in many studies. Here, indicate whether each material, system or method listed is relevant to your study. If you are not sure if a list item applies to your research, read the appropriate section before selecting a response.

Materials & experimental systems

n/a	Involved in the study	
☒	☐	Antibodies
☒	☐	Eukaryotic cell lines
☐	☒	Palaeontology and archaeology
☒	☐	Animals and other organisms
☒	☐	Human research participants
☒	☐	Clinical data
☒	☐	Dual use research of concern

Methods

n/a	Involved in the study	
☒	☐	ChIP-seq
☒	☐	Flow cytometry
☒	☐	MRI-based neuroimaging

Palaeontology and Archaeology

Specimen provenance	The samples/ceramics studied come from the site of Saqqara in Egypt (excavation directed by Ramadan B. Hussein, co-author of the paper). They are from the 2018 and 2019 excavation campaigns.
Specimen deposition	The pottery studied are currently stored at the Saqqara site (Deposit room-magazine) in Egypt. The powder samples taken from the pottery and the organic residues extracted from them are currently stored at the National Research Centre (NRC), Chromatographic Laboratories Network, Giza, Egypt.
Dating methods	*If new dates are provided, describe how they were obtained (e.g. collection, storage, sample pretreatment and measurement), where they were obtained (i.e. lab name), the calibration program and the protocol for quality assurance OR state that no new dates are provided.*

☐ Tick this box to confirm that the raw and calibrated dates are available in the paper or in Supplementary Information.

Ethics oversight	No ethical approval or guidance was required. We followed the commonly accepted and published protocols in the Organic Residue Analyses field.

Note that full information on the approval of the study protocol must also be provided in the manuscript.
